# Comprehensive Review of Open-Source Fundus Image Databases for Diabetic Retinopathy Diagnosis

**DOI:** 10.3390/s25185658

**Published:** 2025-09-11

**Authors:** Valérian Conquer, Thomas Lambolais, Gustavo Andrade-Miranda, Baptiste Magnier

**Affiliations:** 1 IMT Mines Ales, 30100 Alès, France; gustavo.andrade-miranda@mines-ales.fr; 2 EuroMov Digital Health in Motion, University Montpellier, IMT Mines Ales, 30100 Alès, France; 3 Service de Médecine Nucléaire, Centre Hospitalier Universitaire de Nîmes, Université de Montpellier, 30000 Nîmes, France

**Keywords:** diabetic retinopathy, fundus image, databases, computer science image analysis

## Abstract

Databases play a crucial role in training, validating, and comparing AI models for detecting retinal diseases, as well as in clinical research, technology development, and healthcare professional training. Diabetic retinopathy (DR), a common diabetes complication, is a leading cause of vision impairment and blindness worldwide. Early detection and management are essential to prevent irreversible vision loss. Fundus photography, known for being economical and non-contact, is a widely applicable gold standard method that offers a convenient way to diagnose and grade DR. This paper presents a comprehensive review of 22 open-source fundus retinal image databases commonly used in DR research, highlighting their main characteristics and key features. Most of these datasets were released between 2000 and 2022. These databases are analyzed through an in-depth examination of their images, enabling objective comparison using color space distances and Principal Component Analysis (PCA) based on 16 key statistical features. Finally, this review aims to support informed decision-making for researchers and practitioners involved in DR diagnosis and management, ultimately improving patient outcomes.

## 1. Introduction

Diabetes is a complex disease that can have autoimmune, genetic, or acquired causes, impairing the body’s ability to regulate blood sugar, often involving problems with insulin production, insulin action, or both. This hormone is crucial for enabling glucose uptake into cells, thereby regulating blood sugar levels [1]. Persistent high blood sugar levels can result in the production of acetone, which reflects metabolic disturbances associated with diabetes and is often observed in patients with complications such as nephropathy and coronary artery disease. This chronic hyperglycemia leads to biochemical and microvascular changes in the retina and consequently to diabetic retinopathy [2].

**Figure 1:**
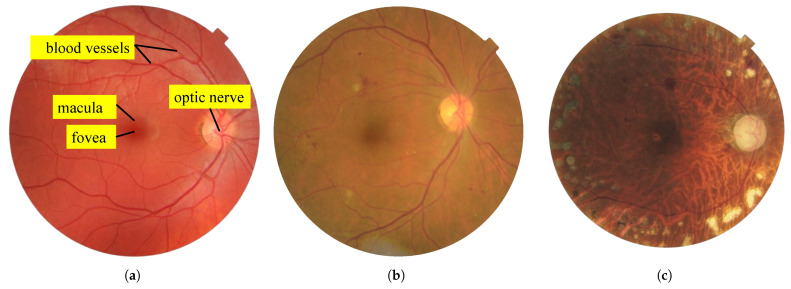
Examples of fundus images. (**a**) Fundus image of a healthy subject showing anatomical structures [10]. (**b**) Fundus image of an unhealthy subject showing signs of non-proliferative diabetic retinopathy (NPDR) [11]. (**c**) Fundus image of an unhealthy subject showing signs of proliferative diabetic retinopathy (PDR) [12].

Notably, diabetic retinopathy (DR), a microvascular complication of diabetes, occurs when high blood sugar levels damage the blood vessels in the retina [3,4]. This light-sensitive layer, essential to vision, can be damaged and eventually lead to blindness if not treated promptly [5]. Globally, DR affects approximately one-third of all individuals with diabetes, with vision-threatening stages present in approximately 10% of diabetic patients [6]. Clinically, the progression of DR is typically classified into two main stages [7]:

- **Non-proliferative diabetic retinopathy (NPDR)**: This stage is marked by weakened, bulging, or leaking retinal blood vessels, resulting in microaneurysms, hemorrhages, and fluid accumulation. It can lead to retinal edema and the formation of exudates. As ischemia progresses, additional vascular occlusions worsen the oxygen deprivation in the retina. An example is displayed in Figure 1b.
- **Proliferative diabetic retinopathy (PDR)**: Characterized by neovascularization as an attempt to compensate for hypoxia, these fragile new blood vessels are prone to bleeding, leading to serious complications such as vitreous hemorrhage, retinal detachment, and neovascular glaucoma. An example is presented in Figure 1c.

Symptoms of DR often develop gradually and may initially go unnoticed. Common symptoms include blurred vision, dark spots, difficulties with color perception, and sudden vision loss. Several risk factors influence the development and progression of DR, including the duration of diabetes, blood sugar control, hypertension, dyslipidemia, pregnancy, and smoking. Effective treatment options depend on the severity and stage of the disease and extend beyond ocular interventions such as laser photocoagulation, intravitreal injections, and vitrectomy [5,8,9]. Preventive care and management strategies are equally crucial and include regular eye examinations, strict glycemic control, management of comorbid conditions, and healthy lifestyle changes. A key diagnostic tool in DR screening and monitoring is the acquisition of high-resolution retinal photographs of the back of the eye, known as fundus images. These images reveal important anatomical structures such as the retina, optic disc, macula, and blood vessels. Higher-resolution images exhibit greater detail, thereby enabling the detection of early signs of DR and other ocular pathologies. As shown in Section 4.5.2, the detection of such pathologies can be challenging due to the small size of their lesions. As illustrated in Figure 1a, several important anatomical structures can be observed in a fundus image:

- **Blood vessels**: These carry blood to and from the retina, appearing as fine lines in fundus images.
- **Macula**: Oval-shaped, yellowish area near the center of the retina responsible for detailed central vision; it appears darker in retinal images.
- **Fovea**: Located within the macula, the fovea has the highest density of cones in the retina.
- **Optic nerve**: Transmits visual information to the brain, appearing as a large spot positioned medial to the macula.

The anatomical features visible in fundus images are essential for diagnosing and monitoring DR, as they provide critical visual information that complements clinical symptoms and risk factors. In this context, the present paper focuses on publicly available fundus image databases, which serve as reference standards for clinical evaluation and are vital for training artificial intelligence models and conducting research in automated retinal disease detection. The paper is organized as follows: Section 2 presents the main retinal imaging techniques, particularly those used for acquiring fundus photographs; Section 3 introduces how these images are leveraged for disease classification and severity assessment; Section 4 reviews the most widely used public retinal imaging databases, detailing their structure and intended applications; and finally, Section 5 offers a discussion of current challenges and perspectives, concluding the study.

In recent years, numerous studies have investigated diabetic retinopathy through segmentation, classification, and generalization tasks using fundus images. Comprehensive surveys and benchmarks have been published to evaluate the performance of various algorithms and to highlight the limitations of generalization across different datasets and disease stages [13,14,15]. Recent works have focused on cross-dataset and domain generalization to improve model robustness and transferability [16,17,18], reflecting the growing interest in designing models that can adapt to variability in image quality, lesion appearance, and annotation styles across real-world clinical settings.

In this context, the present paper focuses on publicly available fundus image databases, which serve as reference standards for clinical evaluation and are vital for training artificial intelligence models and conducting research in automated retinal disease detection.

## 2. Retinal Picturing Methods

Accurate imaging is a fundamental step in diagnosing and monitoring retinal diseases, including diabetic retinopathy (DR), age-related macular degeneration (AMD), and glaucoma. Four primary retinal imaging methods are identified in clinical practice, each with distinct procedural characteristics and diagnostic capabilities. These methods lead to the acquisition of fundus images, which are later analyzed for pathological signs. Two of the techniques require pharmacological pupil dilation using mydriatic eye drops to provide an unobstructed view of the retina, while the other two are non-mydriatic and allow for easier deployment in screening programs. Importantly, these imaging modalities are not limited to DR but are widely used across ophthalmology for general retinal evaluation and disease monitoring [19]. Traditional examination techniques such as indirect ophthalmoscopy and slit-lamp biomicroscopy (with lenses like the Volk or Goldmann lens) remain essential in clinical settings for detailed visualization of the eye, particularly the periphery, and often require mydriasis for optimal performance [20,21].

### Retinal Photography

Retinal photography is a non-invasive imaging method used to acquire high-resolution digital images of the eye using a specialized fundus camera. It is a cornerstone modality in both clinical and screening settings for the documentation and analysis of retinal health. Standard retinal photographs typically cover a field of view ranging from 30° to 50°, centered on the macula or optic disc, although extended protocols such as the ETDRS 7-field system or widefield (90°–120°) and ultrawidefield imaging (up to 200°) may be used to capture more peripheral retinal areas [22,23]. Fundus cameras may rely on conventional color photography or scanning laser ophthalmoscopy (SLO), each with specific characteristics in terms of resolution, contrast, and spectral channels (e.g., Optos ultrawidefield devices use only red and green wavelengths).

Retinal photographs are typically acquired within 1 to 2 min per eye and, in many cases, without requiring pharmacological pupil dilation. However, dilation may be necessary in patients with small pupils or media opacities to ensure sufficient image quality. The captured images are stored for longitudinal monitoring, enabling assessment of disease progression or treatment response in chronic conditions such as DR, glaucoma, and age-related macular degeneration.

This imaging modality is particularly well suited to automated analysis, including AI-based diagnostic support, which may operate in near real time or be integrated into delayed-reading workflows. In such workflows, images are captured at one location (e.g., by trained technicians in a primary care setting) and interpreted later by specialists, enabling efficient deployment of mass screening programs and telemedicine platforms [24].

## 3. Disease Classification and Assessment

**Figure 2:**
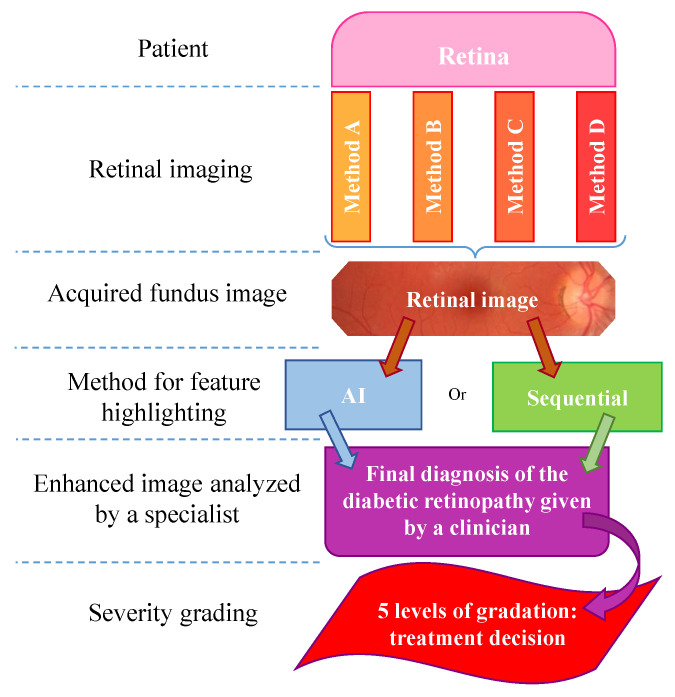
Flowchart detailing the stages of diabetic retinopathy diagnosis from fundus image acquisition to severity classification.

Once retinal images have been successfully acquired, they can be analyzed to identify and grade the patient’s ocular condition. This analysis can be performed manually by an ophthalmologist or other qualified professionals such as optometrists, general practitioners, or certified retinal graders, using computer-aided diagnostic tools. In recent years, automated methods—particularly those based on deep learning—have gained significant traction for DR detection, as outlined in the diagnostic flowchart in Figure 2. These methods generally fall into two categories: learning-based approaches and sequential (rule-based) processes.

### 3.1. Learning-Based Methods

These approaches analyze retinal images by comparing them to large annotated datasets containing known pathological features. These systems learn complex visual patterns and continuously improve as they are exposed to new data, thereby enhancing both sensitivity and specificity in DR detection. Despite their strong performance, these models require significant computational resources and large volumes of high-quality labeled images for training—ideally with the highest possible image quality, although this is not always achievable in practice. Popular deep learning architectures for this task include convolutional neural networks (CNNs) and transformer-based models, which have demonstrated high diagnostic accuracy in different tasks [25,26].

### 3.2. Sequential (Rule-Based) Processing

Alternatively, sequential processing methods rely on predefined rules and image-processing steps. These include filtering and segmentation procedures designed to isolate relevant retinal structures while avoiding confusion between pathological signs and normal anatomical features such as the optic disc, macula, or fovea. These steps often include masking of the optic disc and targeted region extraction prior to classification. These methods are computationally efficient and do not require large training datasets. However, they lack adaptability, are less robust to image variability, and do not improve with continued use, limiting their generalization and scalability.

A common challenge in both methods is the poor quality of fundus images due to issues such as improper lighting, motion blur, or media opacities [27]. These limitations can hinder the visibility of key features and reduce diagnostic performance. To address this, image enhancement techniques—particularly those that are contrast-sensitive—are often employed as a preprocessing step. Such techniques improve the visibility of microaneurysms, hemorrhages, and exudates, thereby aiding in more accurate feature segmentation and classification [28].

### 3.3. DR Severity Grading

**Figure 3:**
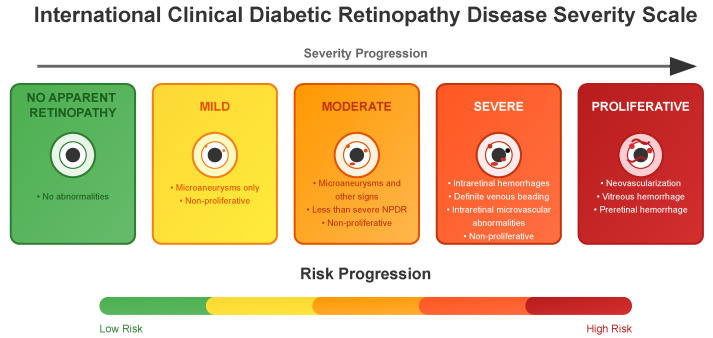
Diagram detailing the five standardized classification levels of the International Clinical Diabetic Retinopathy disease severity scale with a list of corresponding clinical manifestations and risk progression. For a complete definition, refer to Wilkinson et al. [29].

To facilitate diagnosis and clinical decision-making, diabetic retinopathy is typically classified into five standardized levels of severity: the International Clinical Diabetic Retinopathy (ICDR) severity scale. These levels, and their corresponding numbers commonly used in datasets, are detailed in Figure 3 and below [29]:

- **No apparent retinopathy (Level 0)**: No abnormalities are present. Patients typically continue with routine diabetes management and regular eye examinations.
- **Mild (Level 1)**: Microaneurysms only and non-proliferative. Increased monitoring is recommended, and patients may be advised on better glycemic and blood pressure control to prevent progression.
- **Moderate (Level 2)**: More than just microaneurysms but less than severe non-proliferative diabetic retinopathy. The patient might need more frequent eye exams, and the healthcare team may consider medical interventions to manage diabetes more aggressively.
- **Severe (Level 3)**: A significant worsening of the condition and a high risk of progression to proliferative DR, presence of signs such as intraretinal hemorrhages, definite venous beading, or prominent intraretinal microvascular abnormalities.
- **Proliferative State (Level 4)**: The most advanced and sight-threatening stage with one or more of the following: neovascularization, vitreous or preretinal hemorrhage. Urgent treatment is required, which may include intravitreal injections, laser therapy, or vitrectomy to prevent permanent vision loss.

The classification and assessment of diabetic retinopathy (DR) from fundus images depend critically on image quality, the chosen diagnostic methodology, and the precise detection of signs. Learning-based methods, particularly those employing deep learning, have demonstrated strong potential for automated analysis; however, their performance is largely dependent on the availability of large-scale, well-annotated image datasets. Accordingly, the identification and use of publicly available fundus image databases are essential for enabling reliable classification aligned with standardized grading protocols. Such classification is a key component in supporting timely clinical decision-making and improving patient outcomes. The following section reviews the principal public datasets that facilitate the development and validation of automated diagnostic systems for DR.

## 4. Retinal Image Databases for DR Research

This section provides an overview of retinal image databases used in diabetic retinopathy (DR) research that are publicly available for research purposes. In particular, it highlights 22 datasets, each with distinct characteristics, including image count, resolution, format, geographic origin, publication year, and the availability of expert annotations.

### 4.1. Key Points

- **Dataset Variability:** There is significant variability in dataset sizes, ranging from 28 images in AGAR300 to 88,702 images in EyePACS. Image resolutions vary significantly, potentially affecting the reliability and accuracy of subsequent analyses.
- **Image Formats:** The datasets employ a range of standard image formats—JPEG, PNG, TIFF, and BMP—each with varying compression characteristics that affect file size and storage efficiency, as well as image quality, especially the preservation of fine details and the presence of compression artifacts.
- **Labels and Annotations:** A key aspect of dataset utility for deep learning is the availability of either image-level labels or pixel-level annotations, which correspond to two distinct use cases. Labels typically refer to DR severity grades (e.g., “moderate NPDR”, “proliferative DR”) and are used in classification tasks. Annotations, in contrast, provide lesion-level localization (e.g., masks for microaneurysms, exudates, hemorrhages) and are used in detection or segmentation tasks. Most deep learning models rely only on labels for severity classification, while others focus on localizing lesions regardless of severity grade. Some datasets offer both, enabling hybrid approaches. Anatomical landmarks such as the optic disc and macula may also be annotated to increase clinical relevance.
- **Data Distribution:** The distribution of patients affected and unaffected by DR differs markedly between datasets. While large-scale datasets like EyePACS include many unaffected patients, others like **DRiDB** and **AGAR300** focus exclusively on DR patients. Such variability affects model training and may introduce bias if class imbalance is not carefully managed.
- **Grading Systems:** The grading systems for disease severity are not consistent across datasets. For example, **Messidor** uses its own grading system based on the number of microaneurysms, hemorrhages, and neovascularization, while others use the International Clinical Diabetic Retinopathy Scale.
- **Annotated Areas:** Datasets provide annotations for various anatomical characteristics and signs, such as blood vessels, exudates, microaneurysms, and hemorrhages. The annotation formats vary, with some datasets providing segmentation masks and others using XML documents. Clarifying whether these annotations are intended for lesion detection or severity classification is essential for selecting appropriate datasets for model development.
- **Color Analysis:** The section includes an analysis of color histograms and statistical features for each dataset, showing differences and similarities in color distribution and image quality.
- **Visualization and Analysis:** Techniques like Multi-Dimensional Scaling (MDS) and Principal Component Analysis (PCA) are used to visualize and analyze the similarities and differences between datasets based on color histograms and other features.

Overall, this section emphasizes the importance of selecting (or combining) appropriate datasets for specific research objectives and the need for precise and consistent annotations to develop robust machine learning models for DR detection.

### 4.2. General Overview

Table 1 provides an overview of the main fundus image databases used in diabetic retinopathy research. A total of 22 datasets are listed, including:

- **AGAR300**: Annotated Germs for Automated Recognition [30]
- **APTOS**: Asia Pacific Tele-Ophthalmology Society [31]
- **BRSET**: Brazilian Multilabel Ophthalmological Dataset [32]
- **CHASE DB1**: CHASE DataBase 1 [33]
- **DDR**: Dataset for Diabetic Retinopathy [34]
- **DiaRetDB0**: Diabetic Retinopathy DataBase 0 [35]
- **DiaRetDB1**: Diabetic Retinopathy DataBase 1 [36]
- **DR HAGIS**: Diabetic Retinopathy, Hypertension, Age-related Macular Degeneration and Glaucoma Images [37]
- **DRiDB**: Diabetic Retinopathy image DataBase [38]
- **DRIVE**: Digital Retinal Images for Vessel Extraction [39]
- **E-ophtha**: E-ophtha [40]
- **Eye PACS**: Eye Picture Archive Communication System [11]
- **F-DCVP**: Fundus-Data Computer Vision Project [41]
- **HEI-MED**: Hamilton Eye Institute Macular Edema Dataset [42]
- **HRF**: High-Resolution Fundus Segmentation [43]
- **IDRID**: Indian Diabetic Retinopathy Image Dataset [28]
- **JSIEC**: Joint Shantou International Eye Centre [44]
- **Messidor**: Methods to Evaluate Segmentation and Indexing Techniques in the field of Retinal Ophthalmology [45]
- **Messidor2**: Methods to Evaluate Segmentation and Indexing Techniques in the field of Retinal Ophthalmology 2 [45,46]
- **Retina**: Retina [47]
- **ROC**: Online Challenge [48]
- **STARE**: Structured Analysis of the Retina [10]

**Figure 4:**
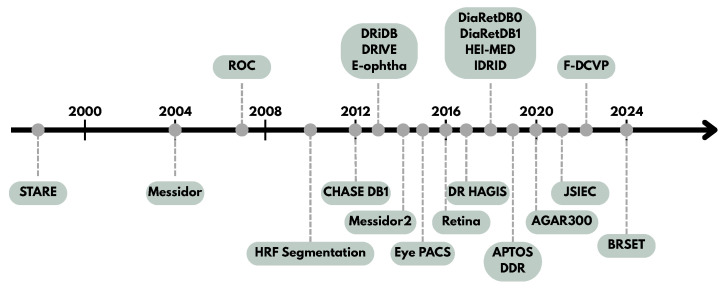
Timeline detailing the date of publication of each dataset.

Each dataset is characterized by the number of images, image resolution, file format, geographic origin, year of publication, and whether the images include annotations. These databases originate from various regions around the world, including India, the UK, Finland, the USA, and France, offering a wide variety of imaging conditions and patient demographics. The databases were also published over a long period, as shown in Figure 4.

**Figure 5:**
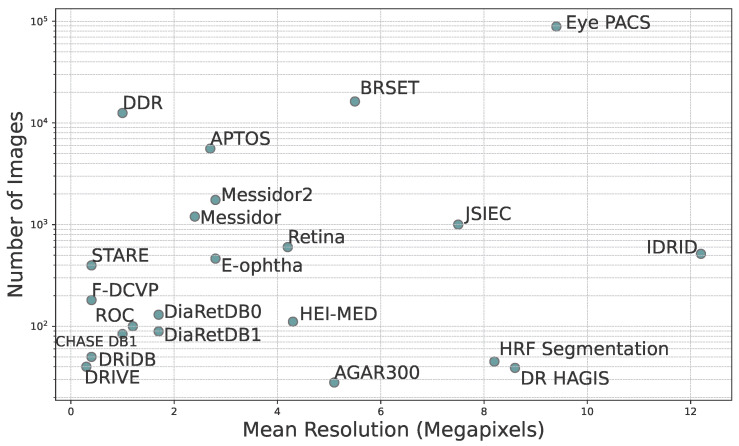
Scatter plot showing the total number of images in each database as a function of mean resolution in megapixels. The mean resolution is the mean of all image resolutions in the dataset.

As previously mentioned, there is significant variation in the size of datasets and images. The second column of Table 1 reports the total number of images and Figure 5 shows a visual comparison of the mean resolution to the number of images in each dataset. Most datasets provide low-resolution images. This means that the image resolution is far below the high-definition (HD) standard of 1280 × 720 pixels and is closer to 640 × 480. High resolution allows for more detailed capture of the anatomical structures of the eye in fundus images, which is crucial for precise and comprehensive analysis of DR features. Fine details such as microaneurysms, exudates, and hemorrhages can be better visualized and assessed in high-resolution images, facilitating early detection of lesions and monitoring their progression over time. This can also contribute to improving the accuracy of image analysis algorithms and automated diagnostic tools used in both research and clinical practice. Image formats vary across datasets and include JPEG, PNG, TIFF, and BMP, with JPEG being the most common. The format—especially JPEG format—can affect image quality, which may in turn influence the reliability of image processing and feature extraction techniques.

Lastly, the availability of image annotations is a critical factor in training and validating machine learning models for DR detection. Most of the datasets listed provide some form of expert annotation, ranging from global severity grades to pixel-level lesion labels. These annotations are essential for supervised learning approaches and are further discussed in subsequent sections.

### 4.3. Data Distribution and Patient Health Status

Another important characteristic of these databases is the proportion of patients without DR they include. A balanced representation of patients with or without DR is critical for training robust image analysis algorithms. A sufficient number of patients without DR not only improves classification accuracy but also reduces model bias and enhances generalization to real-world screening scenarios. An imbalanced dataset, particularly one heavily biased toward pathological cases, can lead to overfitting and poor performance in detecting normal retinas.

**Figure 6:**
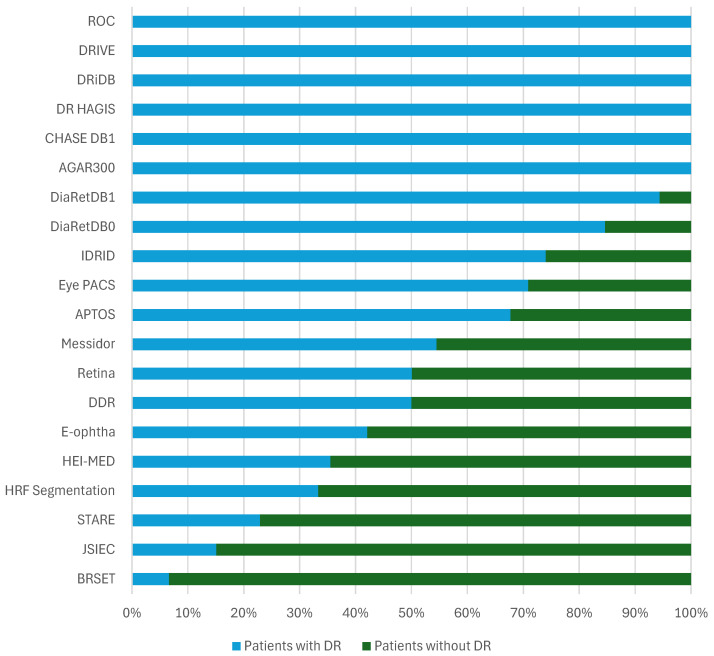
Distribution of patients with DR and without DR across databases. The proportion of patients without DR is compared to all the other fundus images in the dataset that were identified as diseased or not identified. For datasets with training and testing images, in particular, the proportion of patients identified without DR depends on all the images.

The bar chart presented in Figure 6 illustrates the distribution of patients with diabetic retinopathy across various databases, distinguishing between those diagnosed with the DR condition and those without it. This distribution varies significantly between the different databases. In most of them, only patients with DR are provided, which highlights a significant imbalance that can hinder the development of robust and generalizable diagnostic models. The inclusion of patients without DR is closely linked to the specific objective of each database. For instance, **F-DCVP** and **Messidor2** do not provide any information regarding the presence or absence of DR [41]. Therefore, they are not present in Figure 6. While some databases, such as **IDRID** or **DiaRetDB1**, include only a small proportion of patients without DR [28,36]—limiting their usefulness for training algorithms that require balanced datasets—others like **E-ophtha**, **HEI-MED**, and **BRSET** stand out by containing a majority of patients without DR, making them more suitable for developing models capable of distinguishing between DR and other cases [32,40,42].

The **DR HAGIS** and **DRiDB** datasets consist entirely of patients diagnosed with DR [37,38]. These datasets have been specifically curated for the study of the condition, providing a concentrated source of pathological cases. Such datasets are particularly valuable for training, validating, and benchmarking analytical or machine learning models focused on disease detection.

Similarly, the **DRIVE** and **DR HAGIS** datasets only include patients with DR [37,39]. They are especially useful for detailed studies on the manifestation and progression of the disease, supporting the development of specialized diagnostic algorithms and fine-grained lesion analysis.

In contrast, the **APTOS**, **Messidor**, and **E-ophtha** datasets feature a more balanced distribution of patients, including a significant proportion of patients without DR [31,40]. This diversity enhances their suitability for developing and evaluating diagnostic tools capable of distinguishing between DR and non-DR cases, particularly in realistic screening scenarios.

More broadly, the chart highlights the heterogeneous compositions of DR datasets, each designed with distinct research objectives in mind. Datasets predominantly composed of diseased cases are well suited to in-depth pathological studies, such as lesion detection, whereas those that include both cases are essential for developing and validating classification algorithms. This variability underscores the importance of selecting datasets that align with the specific goals and requirements of a given study.

### 4.4. Grading and Annotations

Table 2 provides a comparative overview of various databases used for diabetic retinopathy analysis, emphasizing their key characteristics and differences in annotation formats. Each database is evaluated based on several criteria, including disease severity grading and the presence of annotations for blood vessels, exudates, microaneurysms, and hemorrhages. The comparison highlights the diverse features offered by these databases. They can be broadly categorized into three groups based on their primary focus: blood vessel segmentation, disease severity assessment, and sign-specific annotation.

Note that **F-DCVP** and **Messidor2** are not included in the table, as they do not provide any of the listed annotated features. The **AGAR300** dataset only contains images of patients with microaneurysms but does not provide any annotations [30,41,45,46]. Among the listed datasets, **APTOS**, **DDR**, **Eye PACS**, **JSIEC**, **Messidor**, and **STARE** include disease severity grading [10,11,31,34,44,45]. Although **STARE** includes annotations for multiple lesion types, it does not offer segmentation masks—only severity grading is available. For blood vessel segmentation, **CHASE DB1**, **DR HAGIS**, **DRIVE**, and **HRF Segmentation** are particularly suitable. **DRiDB** and **STARE** also provide segmentation masks of blood vessels [33,37,39,43].

To identify the signs of DR, **DiaRetDB**, **DRiDB**, **E-ophtha**, **HEI-MED**, **IDRID**, and **ROC** are the most relevant datasets. However, the annotation format varies significantly between them. For example, **ROC** annotations are reported in an XML format, where each sign, such as a microaneurysm, is represented by a central position and a radius [48]. Similarly, HEI-MED provides Matlab files. These files are used to generate annotations of the images from the dataset. This approach is generally considered less precise than pixel-wise segmentation masks used in datasets such as **DRiDB** or **IDRID** [28,38].

Some of the datasets fall into multiple categories. For instance, **DRiDB** includes both blood vessels and lesion segmentation, while **IDRID** provides sign-level annotations along with disease severity grading [28,38]. Additionally, several databases contain annotations related to anatomical structures, further enriching the data available for training and evaluation.

In summary, annotation formats vary widely and play a crucial role in detecting the presence, severity, and progression of DR. The precision and consistency of annotations are key to developing robust and well-performing algorithms. Some datasets, such as **DRiDB** and **DiaRetDB**, include annotations from multiple experts—typically five or fewer—which helps improve reliability through consensus [35,36,38]. The annotation format facilitates this process by supporting precise and interpretable labeling.

### 4.5. Analyses of the Annotations

Annotations in retinal image analysis are essential for identifying and grading the severity of diabetic retinopathy (DR). They aid in tracking disease progression and are vital for training machine learning models for automated detection and diagnosis. This section examines the grading criteria used across different datasets, highlighting their key similarities and differences.

#### 4.5.1. Disease Grading

Among the datasets which show disease severity (i.e., **APTOS**, **BRSET**, **DDR**, **Eye PACS**, **IDRID**, **Messidor**, and **STARE**) the grading systems are not always consistent [10,11,28,31,32,34,45]. The **STARE** dataset provides a text document with the labels of the diagnosis. There are two different labels used for DR: “Background Diabetic Retinopathy” and “Proliferative Diabetic Retinopathy”. **Messidor** uses its own grading system: there are no differences between severe NPDR and PDR. These grades range from 0 to 3 and are calculated based on the number of microaneurysms (denoted μA), hemorrhages (*H*), and neovascularization (NV). Disease severity grades are calculated as follows:

$$\begin{array}{ccc} 0\;(\mathrm{Normal}) & : & \left(\mu A=0)\;\mathrm{AND}\;(H=0\right)\\ 1 & : & \left(0<\mu A\leq 5)\;\mathrm{AND}\;(H=0\right)\\ 2 & : & \left((5<\mu A<15)\;\mathrm{OR}\;(0<H<5))\;\mathrm{AND}\;(\mathrm{NV}=0\right)\\ 3 & : & \left(\mu A\geq 15)\;\mathrm{OR}\;(H\geq 5)\;\mathrm{OR}\;(\mathrm{NV}=1\right) \end{array}$$

**Figure 7:**
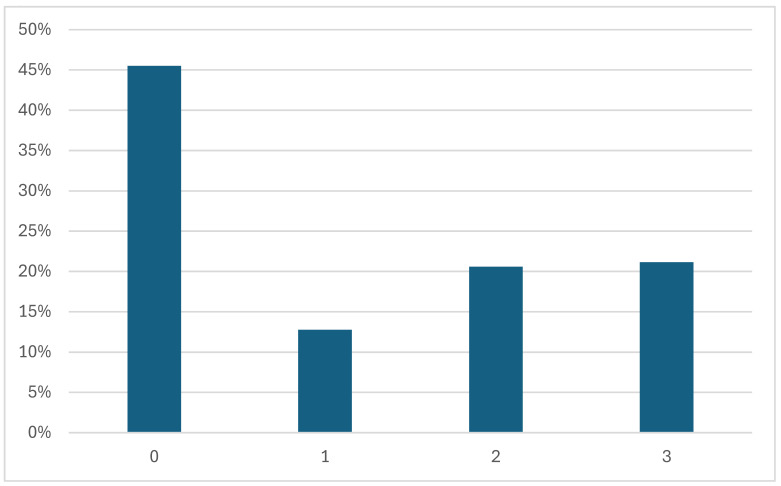
Proportion of each disease grade in the Messidor dataset.

Figure 7 shows the proportion of each grade in the **Messidor** dataset. In other datasets (**DDR**, **IDRID**, **Eye PACS**, **APTOS**, **JSIEC**), these grades range from 0 to 4, and each number corresponds to the progression of the disease, as mentioned in Section 3.3:

- 0—No apparent retinopathy
- 1—Mild
- 2—Moderate
- 3—Severe
- 4—Proliferative DR

**Figure 8:**
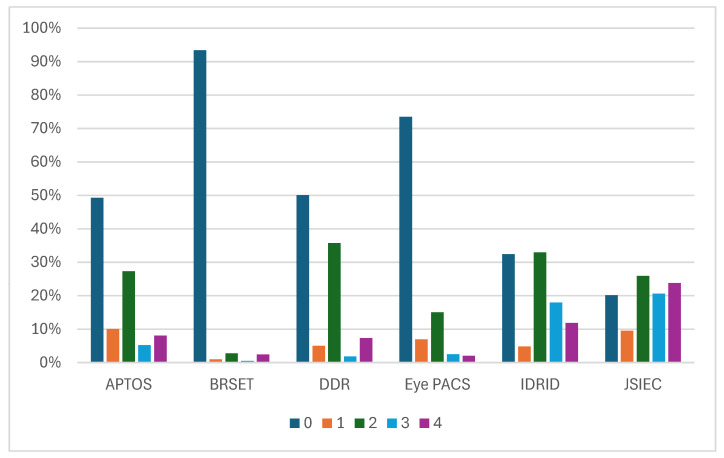
Proportion of each disease grade in **APTOS**, **BRSET**, **DDR**, **IDRID**, **Eye PACS**, and **JSIEC**.

This grading system follows the International Clinical Diabetic Retinopathy Scale, which was introduced earlier (see Figure 3). This scale is used by **APTOS**, **DDR**, **Eye PACS** and **IDRID** [11,28,31,34]. Figure 8 shows a comparison of the proportion of each grade in each dataset that uses the International Clinical DR Scale.

Due to the high quantity of data in **Eye PACS**, that dataset contains many patients without DR [11]. These databases contain few patients with grade 4 DR, which is the rarest case. Another observation is that grade 2 is more prevalent than grade 1 in these four datasets. Note that the **JSIEC** dataset is based on the ICDR severity scale but does not follow it. In **JSIEC**, 0 corresponds to normal eye fundus, 1 to mild NPDR, 2 to moderate NPDR, 3 to severe NPDR and PD,R and 4 to suspected PDR [44]. The dataset sample includes 1000 images, only 189 of which are related to DR, as shown in Figure 6.

#### 4.5.2. Annotated Areas

A wide variety of annotated areas are in these databases, including anatomical characteristics such as the optic disc, blood vessels, and the macula, as well as signs such as hard and soft exudates, hemorrhages, blot hemorrhages, and small red dots.

**Figure 9:**
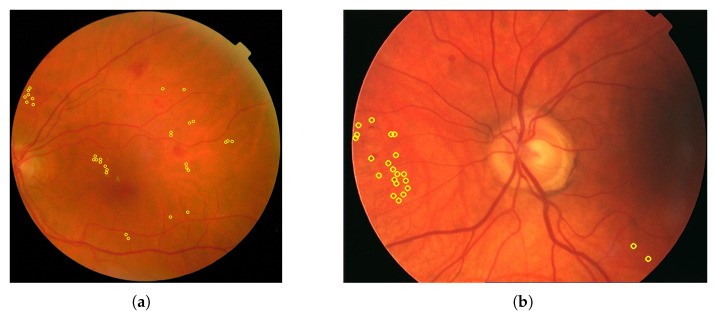
Images from the **ROC** dataset. The microaneurysms are surrounded with yellow. (**a**) Annotations for patient 23. (**b**) Annotations for patient 29.

As it was mentioned before, different formats exist to annotate the fundus images. Most of them are segmentation masks with various binary mask formats such as PNG, BMP, GIF or TIFF. These formats are especially useful for annotating blood vessels. However, there are also TXT documents like in the **STARE** dataset and XML documents like in the **ROC** dataset. TXT documents in the **STARE** dataset contain the number and type of signs for each image but not the position of these signs in the fundus image. In contrast, XML documents provide a position, and the images in Figure 9 of different sizes show the position and the radius of each microaneurysm that has been annotated. Some datasets such as **HEI-MED** and **DiaRetDB0** provide Matlab files that use a screening method to identify signs.

**Figure 10:**
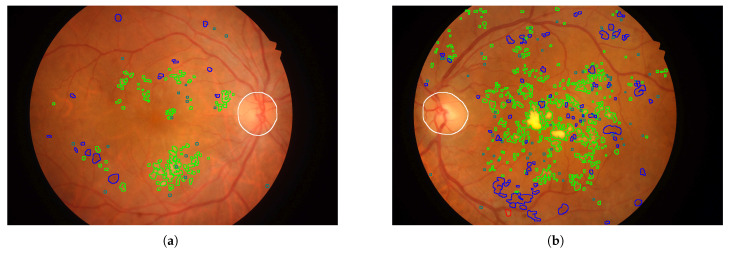
Images from the **IDRID** dataset. Microaneurysms are outlined in gray, hard exudates in green, hemorrhages in blue, optic disc in white, and soft exudates in red. (**a**) Annotations for patient 1. (**b**) Annotations for patient 3.

**Figure 11:**
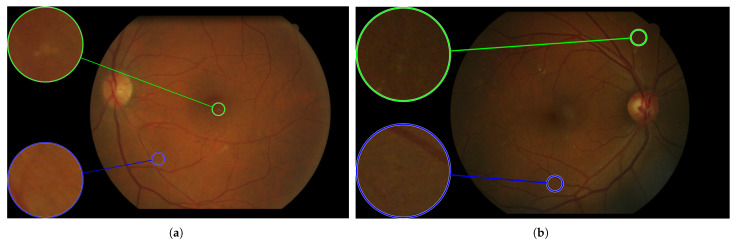
Images of the **E-ophtha** dataset. Exudates are surrounded in green, and the microaneurysms are surrounded in blue. (**a**) Annotations of patient 404. (**b**) Annotations of patient 20305.

**Figure 12:**
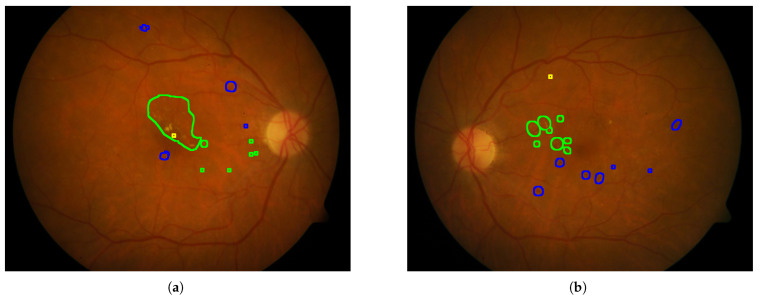
Images of the **DiaRetDB1** dataset with delimited areas corresponding to a threshold of 150 applied to the segmentation mask of each sign. The exudates are outlined in blue, and the microaneurysms are surrounded in yellow. (**a**) Annotations for patient 1. (**b**) Annotations for patient 2.

**Figure 13:**
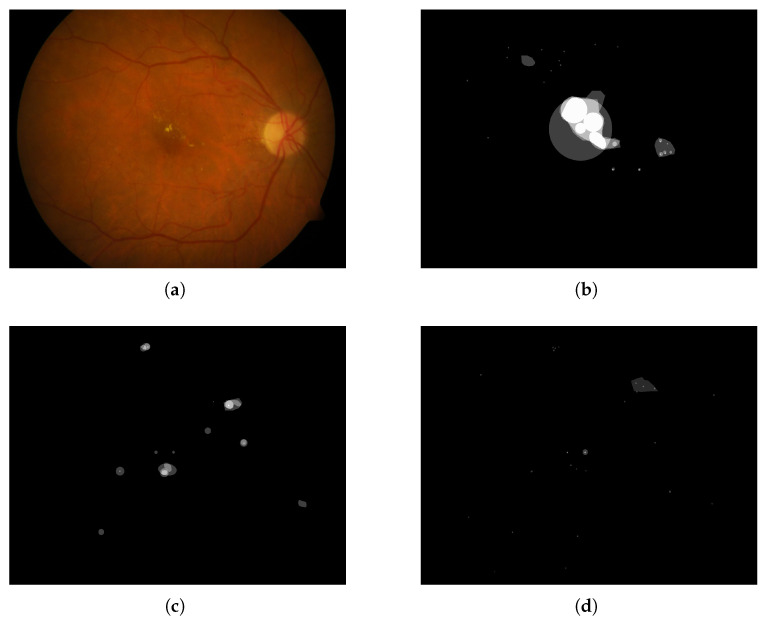
Fundus image from the **DiaRetDB1** dataset with segmentation masks showing annotations from several experts. (**a**) Fundus image of patient 1. (**b**) Hard exudates’ segmentation mask. (**c**) Hemorrhages’ segmentation mask. (**d**) Red small dots’ segmentation mask.

**Figure 14:**
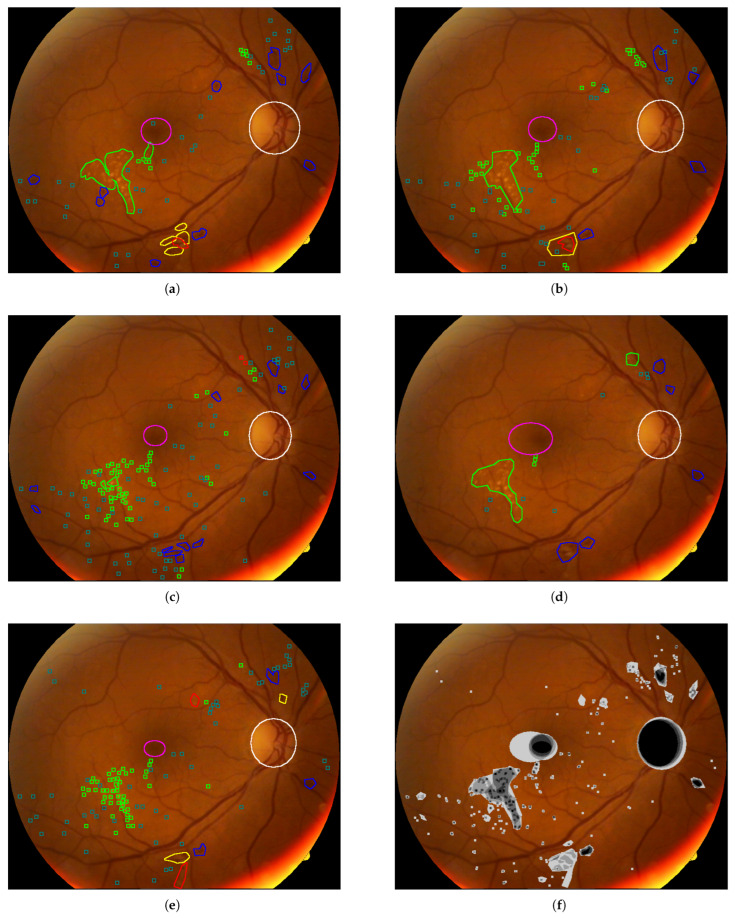
Blot hemorrhages are surrounded in yellow, hard exudates in green, soft exudates in red, hemorrhages in blue, microaneurysm in grayish blue, the macula in magenta, and the optic disc in white. (**a**) Annotations by doctor 1. (**b**) Annotations by doctor 2. (**c**) Annotations by doctor 3. (**d**) Annotations by doctor 4. (**e**) Annotations by doctor 5. (**f**) The intersection over union (IoU) scores for selected structures are as follows: optic disc: 0.69, blot hemorrhages (BH): 0.00, hard exudates (HE): 0.02, hemorrhages (HEM): 0.08, microaneurysms (MA): 0.00027, macula: 0.15, soft exudates (SE): 0.00.

In Figure 10, Figure 11, Figure 12, Figure 13 and Figure 14, images from the **IDRID**, **E-ophtha**, **DiaRetDB1**, and **DRiDB** datasets, are given with segmentation masks of various formats. These segmentation masks were processed in order to isolate and color-code regions that corresponded either to signs of disease or to anatomical features.

In Figure 9, for each image, only one annotator studied these images, and considering that the area covered by each circle is small, the precision is probably not perfect. Figure 10 shows that some annotators provide a very complete analysis of the signs (especially in image (b)), but not all annotators detail the annotations to the same extent, as can be seen in Figure 14 for the **DRiDB** dataset or in Figure 13 [36,38]. The **E-ophtha** dataset also provides segmentation masks for microaneurysms and exudates. Figure 11 shows images from this dataset, and areas of the image where microaneurysms or exudates have been annotated by an expert have been enlarged. This demonstrates the small size of these signs and the importance of having a high-resolution image and several annotators to ensure these details are not missed. In databases that do not feature several annotators per image, it is impossible to know whether the annotations are consensual, as is the case, for example, in the **IDRID** database.

That is why other datasets such as **DiaRetDB1** and **DRiDB** provide segmentation masks of several doctors to establish a consensus and to ensure that these data are as precise as possible [36,38]. The image and the masks below are from the **DiaRetDB1** database. Each segmentation mask represents a different annotated sign from several doctors.

The masks of the **DiaRetDB1** dataset represent the intersection of the different doctor annotations [36]. The whiter the area on the mask, the greater the agreement among doctors on that annotation. In this example, there is strong consensus on hard exudates and hemorrhages, while red small dots show less agreement, as only a few doctors identified that sign. It emphasizes that annotators may disagree about the area covered by a sign or whether a sign is present. The Intersection over Union (IoU) or a defined threshold can help to check if doctors reach a consensus (the IoU is equal to the area of the intersection over the area of the union).

For example, by applying a threshold of 150 (with images encoded with 8 bits) to each segmentation mask, the resulting delimited areas are shown in Figure 12. The Intersection over Union (IoU) shown in Figure 14 was computed for each annotation type of an image from the **DRiDB** dataset. The image (f) of Figure 14 helps to visualize this IoU.

Among the datasets examined in this article, **DRiDB** stands out as the most comprehensive. In addition to sign annotations, it includes detailed markings of anatomical structures such as the optic disc and macula. The patient image presented in Figure 14 was annotated by five different doctors. Each segmentation mask was processed to isolate and color-code specific regions corresponding to either disease signs or anatomical features. This mechanism allows for a clear visual comparison of the differences between annotators. For instance, the annotation in Figure 14c reflects a highly detailed and exhaustive diagnosis, whereas the annotation in Figure 14d highlights broader regions that may not exclusively contain the indicated sign. These differences are further illustrated in Figure 14f, which shows the overlap of annotations across all annotators—darker regions indicate higher agreement.

Thanks to its richness in both pathological and anatomical annotations, the DRiDB dataset is particularly well suited for developing and evaluating computer vision algorithms in medical image analysis.

### 4.6. Annotation Quality and Bias Assessment

Although these datasets benefit from expert annotation, several issues can compromise the consistency and reliability of those labels. First, inter-observer variability remains a crucial point: while some datasets like **IDRID** rely on a single expert annotator—preventing any assessment of consistency—others, such as **DiaRetDB1** and **DRiDB**, provide annotations from multiple clinicians for each image. This enables the derivation of consensus annotations, for example, by intersecting individual masks, and allows for a more quantitative assessment of agreement between observers using metrics like Intersection over Union (IoU), which measures the degree of overlap between independent segmentations. Substantial inter-expert variability in the grading of retinal features is well documented, even among trained specialists, and must be considered in ground-truth generation for automated analyses [49,50,51].

In addition, subjective interpretation and varying annotation precision lead to inconsistencies within the same dataset. Annotators may disagree on the exact delineation of lesion borders, particularly for small or poorly defined features such as red dots or soft exudates. These differences are evident in the variety of masks drawn by different experts in datasets like **DRiDB**, as well as in the use of broad, imprecise circles in datasets like **ROC**, which may not accurately follow lesion contours. Studies show that the agreement for identifying subtle pathologies is typically lower than for more prominent findings [50,51]. Furthermore, it is important to note that annotation errors or ambiguities directly affect not only the training of detection algorithms but also the evaluation itself [52]. When reference annotations are imprecise or inconsistent, common quantitative metrics—such as IoU, Dice coefficient, sensitivity, or specificity—can underestimate the true potential of a model, or conversely, reward predictions that merely match the flaws or biases of the ground truth. As a result, the interpretation of model performance is inseparable from the reliability of the reference annotations.

Class imbalance represents another important source of bias: certain structures (such as hard exudates or the optic disc) are prevalent, whereas others (like soft exudates or blot hemorrhages) may be rare or even absent from many images. This skewed distribution can negatively affect model training, as infrequent classes are often under-represented and thus more challenging for models to learn. Assessing the extent of this imbalance through label distribution graphs or imbalance ratios can provide insight into its potential impact on model performance and generalization [51,53].

Finally, inconsistencies in annotation formats—whether segmentation masks, approximate circles, or text labels—as well as differences in the rigor of quality control (for example, whether expert consensus or independent verification is used) further complicate direct comparison across datasets. Such variation must be thoughtfully considered during training, evaluation, and benchmarking of automated analysis systems to ensure fair and robust comparisons.

#### Color Analysis

The appearance of fundus images is strongly influenced by the type of imaging device used and the principles behind image acquisition. To contextualize, the list below details the imaging devices used across the analyzed datasets, including fundus camera manufacturers and their imaging modalities, when available:

- **AGAR300**: NR
- **APTOS**: NR
- **BRSET**: Canon CR2 camera, Nikon NF5050 retinal camera
- **CHASE DB1**: Nidek NM-200-D fundus camera. The images were captured at a 30-degree field of view with a resolution of 1280 × 960 pixels
- **DDR**: TRC NW48, Nikon D5200, Canon CR 2 cameras
- **DiaRetDB0**: 50-degree field-of-view digital fundus camera
- **DiaRetDB1**: 50-degree field-of-view digital fundus camera
- **DR HAGIS**: TRC-NW6s (Topcon), TRC-NW8 (Topcon), or CR-DGi fundus camera (Canon)
- **DRiDB**: Zeiss VISUCAM 200 fundus camera at a 45-degree field of view
- **DRIVE**: Canon CR5 non-mydriatic 3CCD camera with a 45-degree field of view (FOV). Each image was captured using 8 bits per color plane at 768 × 584 pixels.
- **E-ophtha**: NR
- **Eye PACS**: Centervue DRS (Centervue, Italy), Optovue iCam (Optovue, USA), Canon CR1/DGi/CR2 (Canon), and Topcon NW (Topcon)
- **F-DCVP**: NR
- **HEI-MED**: Visucam PRO fundus camera (ZEISS, Germany)
- **HRF**: CF-60UVi camera (Canon)
- **IDRID**: VX-10 alpha digital fundus camera (Kowa, USA)
- **JSIEC**: NR
- **Messidor**: Topcon TRC NW6 non-mydriatic fundus camera with a 45-degree field of view using 8 bits per color channel and a resolution of 1440 × 960, 2240 × 1488 or 2304 × 1536 pixels.
- **Messidor2**: Topcon TRC NW6 non-mydriatic fundus camera with a 45-degree field of view using 8 bits per color channel and a resolution of 1440 × 960, 2240 × 1488 or 2304 × 1536 pixels.
- **Retina**: NR
- **ROC**: TRC-NW100 (Topcon), TRC-NW200 (Topcon), or CR5–45NM (Canon)
- **STARE**: TRV-50 fundus camera (Topcon)

Most datasets rely on optical fundus photography using traditional retinal cameras (e.g., Topcon, Canon, Zeiss), while scanning laser ophthalmoscopy systems (e.g., Heidelberg, Optos) are not reported in the selected datasets. The captured images generally follow a true-color representation using standard RGB reconstruction, without mention of pseudo-color imaging or multispectral enhancement. When not reported (NR), the dataset documentation provides no details about the acquisition hardware or imaging principles.

For each image in each dataset, the following features were computed for each color channel (red, green, blue) and the grayscale version (the formula used in the Python library OpenCV to convert an image from RGB to grayscale is as follows: Y←0.299·R+0.587·G+0.114·B, where *R*, *G*, and *B* represent the red, green, and blue color channels of the image, respectively; this weighted sum reflects the human eye’s higher sensitivity to green and red light, resulting in a perceptually accurate grayscale representation):

- Mean;
- Minimum;
- Maximum;
- Mean of the local variances (computed using a 5 × 5 window);
- Histograms (blue, green, red, and grayscale).

These features provide both global and local information about image intensity and color distribution. In particular, the local variance helps to quantify texture, edge sharpening, and detail variation in small regions of the image. Additionally, in the figures provided in Supplementary Materials,

1. The **top row** shows box plots for the first four statistical features listed above;
2. The **bottom row** presents the normalized histograms, computed as the average of all image histograms within each dataset.

**Figure 15:**
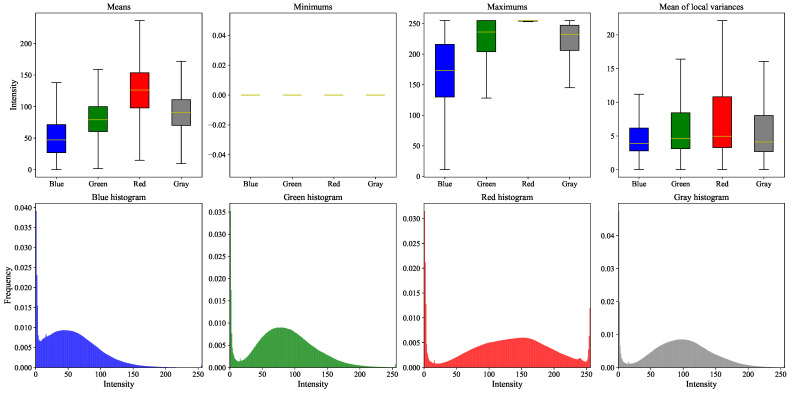
Synthesis of all figures from the Supplementary Materials. Box plots aggregate results across all datasets, with outliers omitted due to their high frequency. Histograms represent the weighted average of all dataset-specific histograms, with weights proportional to the number of images per dataset.

Figure 15 synthesizes all these figures. Note: When the minimum value of a color channel is zero for all images in a dataset, the corresponding box plot is reduced to a single line at zero in the middle of the graph (see Supplementary Materials).

These graphs highlight both the differences and similarities among the datasets in terms of image content. To ensure consistent statistical analysis across datasets, a preprocessing step was applied to isolate the retinal region. When available, fundus masks provided by the datasets were used to crop the images, restricting computations to the region of interest. For datasets lacking such masks, a simple background removal heuristic was employed: only pixels with the exact RGB value #000000 (pure black) were excluded from the analysis. However, this method is not always reliable, as background regions are not uniformly black across all datasets—some may contain near-black values that are not captured by this thresholding, potentially introducing background noise into the statistics. A more common approach to background masking in retinal images is to detect the circular region of interest using Hough transformations or similar techniques such as RANSAC (RANdom SAmple Consensus) [54].

Datasets that include a background mask are **DiaRetDB0**, **DiaRetDB1**, **DR HAGIS**, **DRiDB**, **DRIVE**, and **HRF Segmentation** [35,36,37,38,39,43]. For these six datasets, the box plots of minimum values are more reliable and meaningful, since the background was properly excluded. In contrast, for datasets without explicit masks, the background may vary in intensity and not be purely black. For instance, the box plots of minimum values in the **STARE** dataset reveal outliers above zero across all color channels [10].

**Figure 16:**
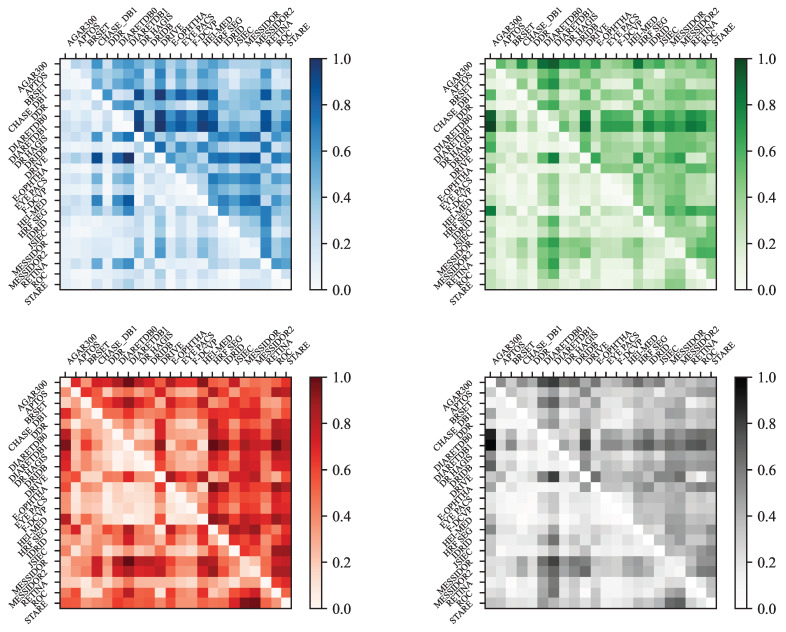
Colored matrices of the Manhattan distance (upper triangle) and the Bhattacharyya distance (lower triangle) between datasets for the blue, green, red, and gray histograms. Note that HRF Seg corresponds to HRF Segmentation.

To better visualize the differences and similarities between datasets, histogram distances are shown in Figure 16, and a Principal Component Analysis (PCA) of the remaining features is presented further.

In Figure 16, each matrix is a square matrix representing the pairwise distances between image datasets based on color histograms. The rows and columns correspond to different datasets, such as **APTOS**, **DRIVE**, **STARE**, and others. There are four matrices in total, each normalized and computed using a different histogram: one for the blue channel, one for the green channel, one for the red channel, and one for the grayscale histogram.

Each matrix displays two types of distances: the upper triangle contains the **Manhattan distances** [55], while the lower triangle shows the **Bhattacharyya distances**. In a given color space, let $H_{1}$ and $H_{2}$ denote the histograms extracted from two different images. Each histogram contains N=256 bins, and for an integer k∈{0,…,255}, $H_{i}\left(k\right)$ represents the value of the *k*th bin of histogram $H_{i}$, where i∈{1,2}. The histograms are normalized so that their bin values sum to one:

$$\sum_{k=0}^{N-1}H_{i}\left(k\right)=1,\;\mathrm{for}\;i=1,2.$$

This normalization allows for a meaningful comparison between histograms using different distance metrics, defined as follows:

- **Manhattan distance (L1 norm)**:
  $$D_{\mathrm{Manhattan}}\left(H_{1},H_{2}\right)=\sum_{k=0}^{N-1}\left|H_{1}\left(k\right)-H_{2}\left(k\right)\right|.$$
- **Bhattacharyya distance** (if histograms are not normalized):
  $$D_{\mathrm{Bhattacharyya}}\left(H_{1},H_{2}\right)=\sqrt{1-\frac{1}{\sqrt{{\bar{H}}_{1}\cdot {\bar{H}}_{2}\cdot N^{2}}}\sum_{k=0}^{N-1}\sqrt{H_{1}\left(k\right)\cdot H_{2}\left(k\right)}},$$
  where
  $${\bar{H}}_{i}=\frac{1}{N}\sum_{k=0}^{N-1}H_{i}\left(k\right),\;\mathrm{for}\;i=1,2.$$

To better interpret the computed distance matrices, we can analyze the patterns that emerge from comparing different datasets. The similarities among datasets that include background masks appear more clearly in the red channel matrix. Nonetheless, the **DRIVE** and **HRF Segmentation** datasets do not seem to follow this pattern. According to Table 2 and the original dataset sources, this discrepancy is likely due to the different purposes of these datasets: **DiaRetDB0**, **DiaRetDB1**, **DR HAGIS**, and **DRiDB** are specifically designed for detecting DR signs, whereas **DRIVE** and **HRF Segmentation** are primarily intended for blood vessel segmentation. Additionally, the **Messidor** dataset appears to differ significantly from the others, particularly in terms of the Bhattacharyya distance in the red channel matrix.

**Figure 17:**
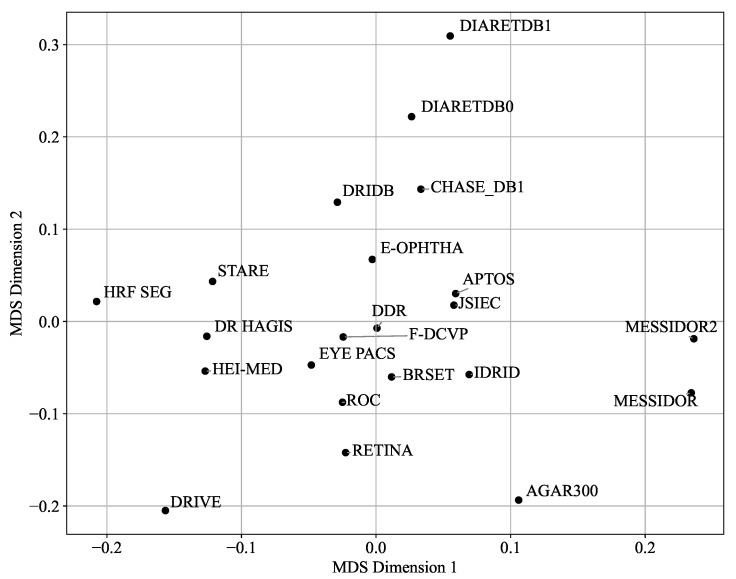
Two-dimensional visualization of dataset similarities (averaged RGB and gray channels, Bhattacharyya distance) using Multi-Dimensional Scaling (MDS).

**Figure 18:**
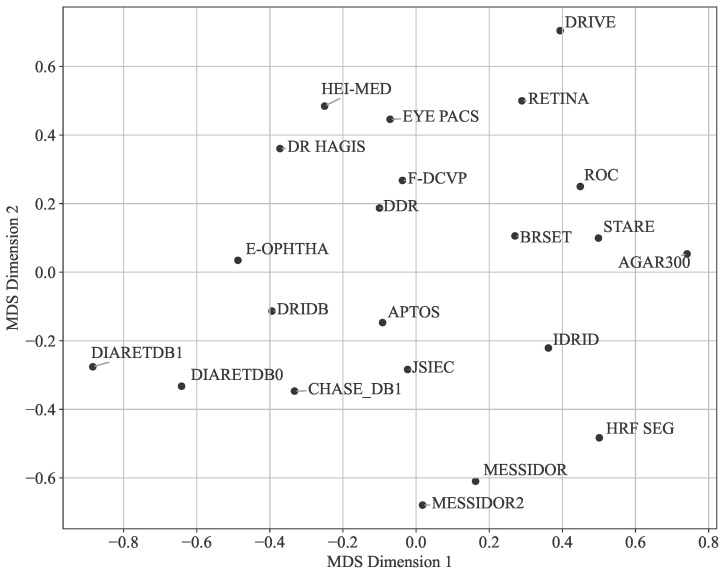
Two-dimensional visualization of dataset similarities (averaged RGB and gray channels, Manhattan distance) using Multi-Dimensional Scaling (MDS).

Figure 17 presents a 2D visualization of the average matrix (averaged over RGB and grayscale channels) based on the Bhattacharyya distance. The visualization was generated using metric Multi-Dimensional Scaling (MDS), which helped to better interpret the relationships depicted by the distance matrices. To realize this reduction in dimension, MDS uses a symmetric matrix $D={\left(d_{ij}\right)}_{1⩽i,j⩽n}$ containing the Bhattacharyya distances between the *n* datasets. Then, the objective of the metric MDS is to find *n* points $y_{1},...,y_{n}\in R^{2}$ that minimize the following stress function:

$$Stress\left(Y\right)=\sum_{i<j}(d_{ij}-\left|\right|y_{i}-y_{j}{\left||\right)}^{2},$$

where $Y={\left[y_{1},...,y_{n}\right]}^{⊤}\in R^{2\times n}$. The same principle was applied to the Manhattan distances in Figure 18. For a more detailed mathematical treatment of MDS algorithms and their applications, see [56].

**Figure 19:**
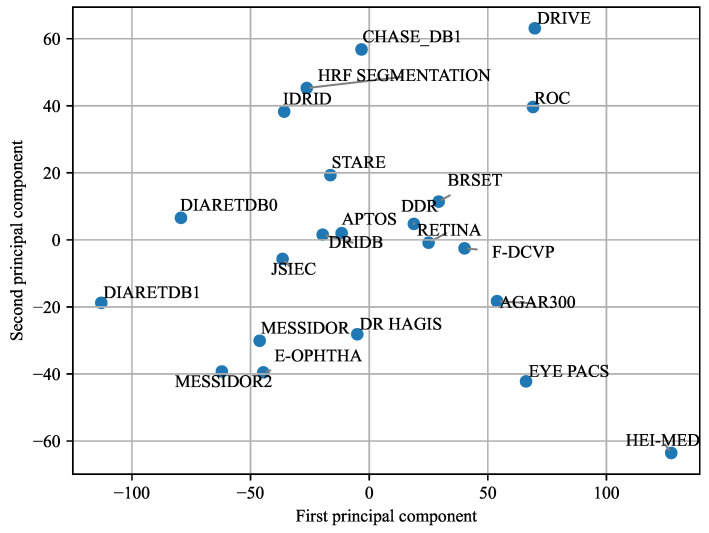
Principal Component Analysis (PCA) of the 22 datasets. The analysis includes 16 components, each corresponding to the median value from the box plots of the different color channels.

According to Figure 17, the **APTOS** and **JSIEC** datasets, the **DDR** and **F-DCVP** datasets, or the **E-ophtha** and **DRiDB** datasets are positioned close to each other, while other datasets such as **AGAR300** and **DRIVE** appear as outliers. The graph in Figure 19 further supports these observations. However, the graph in Figure 18, which uses the Manhattan distance, seems to emphasize the differences even if the outliers remain the same, and the closest datasets are also **APTOS** and **JSIEC**.

While Figure 16 focused on differences between histograms, Figure 19 presents a Principal Component Analysis (PCA) based on the 16 key statistical features derived from the box plots in Supplementary Materials. Specifically, for each image in a dataset, four features were computed for each color channel (red, green, blue, and grayscale): the mean, minimum, maximum, and mean of the local variances. This resulted in 16 features per image. For each dataset, the median value of each of these 16 features was calculated across all images, yielding a single 16-dimensional feature vector per dataset. PCA was then applied to these vectors, and the datasets were visualized in a two-dimensional space using the first two principal components. The PCA plot reveals a clear separation among datasets, suggesting that the selected features captured meaningful differences in image characteristics. However, this approach has certain limitations: it does not consider retinal structures or pathological lesions, and since PCA was applied to aggregated median values, it does not reflect intra-dataset variability.

The PCA results offer valuable insights into the relationships between datasets. When datasets are clustered closely in the PCA plot, it suggests that they share similar characteristics in terms of lighting conditions, color distribution, and overall image quality. In contrast, datasets positioned further apart—such as **AGAR300** and **HEI-MED**—may represent outliers due to differences in acquisition parameters, including exposure, resolution, or preprocessing workflows. Additional factors such as image compression and background segmentation techniques can also contribute to these discrepancies. For instance, **DiaRetDB0** applies a single background mask uniformly across all images, while **DiaRetDB1** assigns a unique mask to each image. This methodological difference may account for the notable divergence observed between these two otherwise comparable datasets [35,36].

Figure 17, Figure 18 and Figure 19 reveal some similarities, such as identifying outliers like **AGAR300**, **DiaRetDB0**, **DiaRetDB1**, and **DRIVE**. However, they differ in terms of which datasets are closest to each other. In Figure 17 and Figure 18, **JSIEC** and **APTOS** are the closest datasets, whereas in Figure 19, **APTOS** is closer to **DRiDB** than to **JSIEC**. Additionally, Figure 19 shows **IDRID** and **HRF Segmentation** as very close, while in Figure 17 and Figure 18, they appear quite different. Neither figure establishes a clear correlation between the main characteristics of the datasets (as summarized in Table 2) and their color histograms or color variations.

The PCA visualization provides a compact representation of inter-dataset differences based on statistical color features. While it effectively reveals outliers—such as **AGAR300**, **HEI-MED**, and **DiaRetDB0**—and highlights certain similarities among datasets, its utility extends beyond basic clustering. Datasets that cluster closely (e.g., **JSIEC**, **APTOS**, **E-OPHTHA**) likely share imaging conditions or preprocessing strategies, which has direct implications for tasks like unsupervised domain adaptation. In contrast, peripheral datasets may present more substantial domain shifts and thus serve as challenging test sets for assessing generalization.

The observed divergence between **DiaRetDB0** and **DiaRetDB1**, despite their institutional similarity, emphasizes how preprocessing choices (e.g., global vs. individual background masking) can have pronounced effects on feature distributions. These technical discrepancies can influence the success of model transferability and domain adaptation. However, the PCA’s reliance on aggregated median features and its exclusion of anatomical or pathological content limits its interpretability for clinical task alignment. Therefore, while useful for dataset comparison, PCA should be supplemented with more task-specific evaluations.

## 5. Conclusions and Discussion

This review highlighted the crucial role of open-source fundus image databases in advancing diabetic retinopathy (DR) diagnosis, particularly in the context of machine learning and automated analysis. The diversity of these datasets—regarding resolution, grading systems, annotations, and patient demographics—offers valuable opportunities for algorithm development, while also posing significant challenges in terms of standardization and model generalization.

A key takeaway is the impact of annotation quality and consistency. Datasets with pixel-level segmentation masks from multiple annotators, such as **DiaRetDB1** and **DRiDB**, offer a higher degree of reliability and are particularly suited to tasks requiring fine-grained lesion detection. Conversely, databases that rely on single-expert annotations or less precise formats (e.g., XML coordinates) may introduce variability that hinders model generalization.

**Figure 20:**
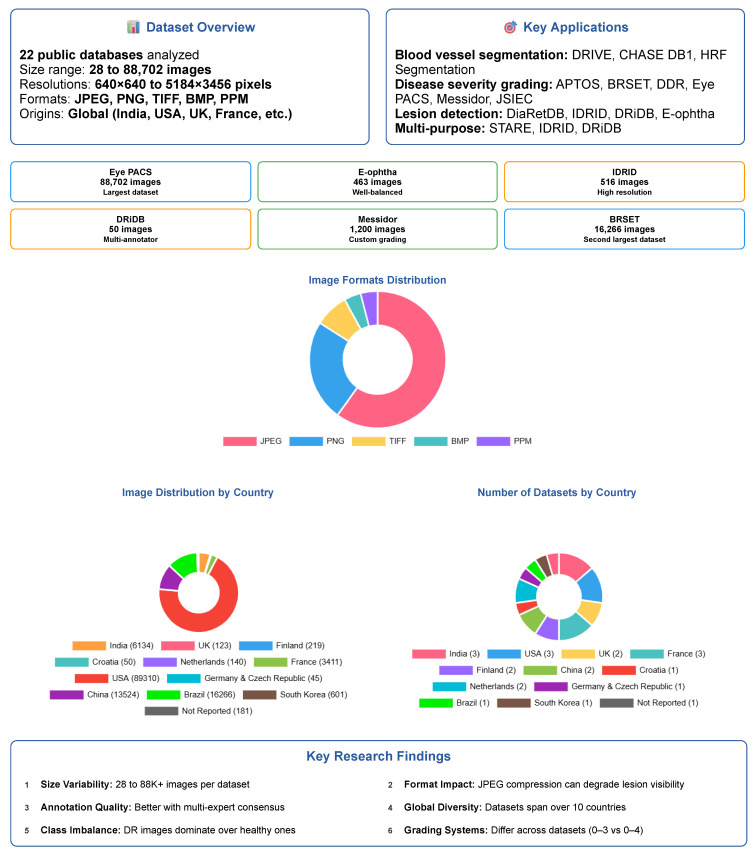
Summary of the paper’s key findings on retinal fundus databases for diabetic retinopathy research; it does not include the color analysis section.

Another important factor is the balance between patients affected and unaffected by DR. While many datasets over-represent DR patients to focus on pathological findings, this can lead to bias in classification models. Datasets like **E-ophtha** and **Eye PACS**, which include a substantial number of patients without DR individuals, are more appropriate for developing robust screening tools aimed at distinguishing between normal and pathological cases. Finally, the color analysis of fundus images reveals significant variability across datasets, which may affect the performance of image enhancement and preprocessing algorithms. The use of fundus masks is recommended to reduce noise and ensure consistency during image processing. The Figure 20 summarizes the paper’s key findings.

### Recommendations

- **For segmentation tasks:** Favor datasets with pixel-level annotations and central positioning in the PCA space (e.g., **DRiDB** and **IDRID** for lesions segmentation, **HRF Segmentation** and **DRIVE** for blood vessel segmentation). Use caution when incorporating outlier datasets like **AGAR300**, as their distinct characteristics may hinder generalization.
- **For classification:** Datasets such as **APTOS** and **EYE-PACS**, which lie near the PCA centroid, may provide a good balance of feature diversity and representativeness for model training. They are also based on the ICDR severity scale, and one of the severity classes is not under-represented.
- **For dataset selection or benchmarking:** Central datasets like **MESSIDOR** and **EYE-PACS** can serve as robust baselines. Outliers such as **HEI-MED** or **DiaRetDB0** can be used to evaluate performance under less-controlled acquisition settings.

To summarize, this review provides a detailed comparative framework for selecting the most appropriate open-source fundus image database based on specific research or clinical objectives. As AI-driven DR diagnosis tools continue to evolve, standardization in grading systems, annotation formats, and data diversity will be essential to ensure their reliability, interpretability, and clinical acceptance. Future efforts should focus on creating harmonized datasets with detailed, consensus-driven annotations and broad demographic representation to support the development of equitable and accurate diagnostic systems.

## Figures and Tables

**Table 1:** Overview of the main publicly available retinal fundus image databases used in diabetic retinopathy research (DR), with publication years ranging from 2000 to 2022. The table summarizes key characteristics of each dataset, including the total number of images, image resolution, file format, geographic origin, year of publication, and availability of expert annotations. These datasets vary widely in size, image quality, and annotation detail, reflecting diverse acquisition settings and research purposes. If the dataset itself contains different resolutions, the range of resolutions is provided. Here, #Images represents the total number of images included in the dataset. All datasets were downloaded before 30 May 2025, except for BRSET, DDR and Messidor2, which were downloaded before 31 July 2025.

Name	#Images	Resolution	Format	Geographical Origin		Year of Publication	DR and Other Diseases	Annotations
AGAR300	28	from 1020 × 1225 to 3696 × 2448	JPEG	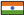	India	2020		No
APTOS	5590	from 474 × 358 to 4288 × 2848	JPEG	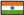	India	2019		No
BRSET	16,266	from 951 × 874 to 2420 × 1880	PNG, JPEG	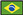	Brazil	2024		Yes
CHASE DB1	84	999 × 960	JPEG, PNG	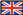	UK	2012		Yes
DDR	12,524	from 512 × 512 to 5184 × 3456	JPEG	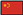	China	2019		No
DiaRetDB0	130	1500 × 1152	PNG	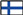	Finland	2018		Yes
DiaRetDB1	89	1500 × 1152	PNG	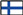	Finland	2018		Yes
DR HAGIS	39	from 2816 × 1880 to 4752 × 3168	JPEG, PNG	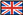	UK	2017	✓	Yes
DRiDB	50	720 × 576	BMP	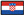	Croatia	2013		Yes
DRIVE	40	768 × 584	JPEG	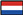	Netherlands	2013		Yes
E-ophtha	463	from 1440 × 960 to 2544 × 1696	JPEG	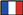	France	2013		Yes
Eye PACS	88,702	640 × 640	JPEG	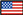	USA	2015		Yes
F-DCVP	181	640 × 640	JPEG	?	?	2022		No
HEI-MED	111	4752 × 3168	JPEG	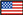	USA	2018		Yes
HRF Segmentation	45	3504 × 2336	JPEG	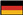 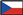	Germany and Czech Republic	2010	✓	Yes
IDRID	516	4288 × 2848	JPEG	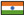	India	2018		Yes
JSIEC	1000	from 768 × 576 to 3152 × 3000	JPEG	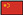	China	2021	✓	No
Messidor	1187	2240 × 1488	TIFF	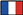	France	2004		Yes
Messidor2	1748	2240 × 1488	TIFF	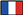	France	2014		No
Retina	601	from 1848 × 1224 to 2592 × 1728	PNG	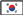	South Korea	2016	✓	No
ROC	100	from 768 × 576 to 1394 × 1391	JPEG	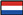	Netherlands	2007		Yes
STARE	397	700 × 605	PPM	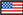	USA	before 2000	✓	Yes

**Table 2:** Summary of diabetic retinopathy database attributes, including disease classification labels, vascular structures, lesion types, and annotation details.

Name	Labels	Blood Vessels	Lesions			Annotation Format
			Exudates	Microaneurysms	Hemorrhages	
AGAR300				✓		None
APTOS	✓					None
BRSET	✓				✓	None
CHASE DB1		✓				PNG
DDR	✓					None
DiaRetDB0			✓	✓	✓	PNG
DiaRetDB1			✓	✓	✓	PNG
DR HAGIS		✓				PNG
DRiDB		✓	✓	✓	✓	BMP
DRIVE		✓				GIF
E-ophtha			✓	✓		PNG
Eye PACS	✓					None
HEI-MED			✓		✓	Matlab files
HRF Segmentation		✓				TIFF
IDRID	✓		✓	✓	✓	TIFF
JSIEC	✓					None
Messidor	✓					None
ROC				✓		XML
STARE	✓	✓	✓	✓	✓	TXT

## Supplementary Materials

The following supporting information can be downloaded at: https://www.mdpi.com/article/10.3390/s25185658/s1, Figure S1: Average color histograms (RGB and grayscale) and box plots of key statistical features for all images in the AGAR300 dataset. Figure S2: Average color histograms (RGB and grayscale) and box plots of key statistical features for all images in the APTOS dataset. Figure S3: Average color histograms (RGB and grayscale) and box plots of key statistical features for all images in the BRSET dataset. Figure S4: Average color histograms (RGB and grayscale) and box plots of key statistical features for all images in the CHASE DB1 dataset. Figure S5: Average color histograms (RGB and grayscale) and box plots of key statistical features for all images in the DDR dataset. Figure S6: Average color histograms (RGB and grayscale) and box plots of key statistical features for all images in the DiaRetDB0 dataset. Figure S7: Average color histograms (RGB and grayscale) and box plots of key statistical features for all images in the DiaRetDB1 dataset. Figure S8: Average color histograms (RGB and grayscale) and box plots of key statistical features for all images in the DR HAGIS dataset. Figure S9: Average color histograms (RGB and grayscale) and box plots of key statistical features for all images in the DRiDB dataset. Figure S10: Average color histograms (RGB and grayscale) and box plots of key statistical features for all images in the DRIVE dataset. Figure S11: Average color histograms (RGB and grayscale) and box plots of key statistical features for all images in the E-Ophtha dataset. Figure S12: Average color histograms (RGB and grayscale) and box plots of key statistical features for all images in the Eye PACS dataset. Figure S13: Average color histograms (RGB and grayscale) and box plots of key statistical features for all images in the F-DCVP dataset. Figure S14: Average color histograms (RGB and grayscale) and box plots of key statistical features for all images in the HEI MED dataset. Figure S15: Average color histograms (RGB and grayscale) and box plots of key statistical features for all images in the HRF Segmentation dataset. Figure S16: Average color histograms (RGB and grayscale) and box plots of key statistical features for all images in the IDRID dataset. Figure S17: Average color histograms (RGB and grayscale) and box plots of key statistical features for all images in the JSIEC dataset. Figure S18: Average color histograms (RGB and grayscale) and box plots of key statistical features for all images in the MESSIDOR dataset. Figure S19: Average color histograms (RGB and grayscale) and box plots of key statistical features for all images in the MESSIDOR2 dataset. Figure S20: Average color histograms (RGB and grayscale) and box plots of key statistical features for all images in the Retina dataset. Figure S21: Average color histograms (RGB and grayscale) and box plots of key statistical features for all images in the ROC dataset. Figure S22: Average color histograms (RGB and grayscale) and box plots of key statistical features for all images in the STARE dataset.

## Abbreviations

The following abbreviations are used in this manuscript:

AI	Artificial Intelligence
DR	Diabetic Retinopathy
NPDR	Non-Proliferative Diabetic Retinopathy
NR	Not Reported
PDR	Proliferative Diabetic Retinopathy
PCA	Principal Component Analysis
ICDR	International Clinical Diabetic Retinopathy
AGAR	Annotated Germs for Automated Recognition
APTOS	Asia Pacific Tele-Ophthalmology Society
BRSET	Brazilian Multilabel Ophthalmological Dataset
DDR	Dataset for Diabetic Retinopathy
DiaRetDB	Diabetic Retinopathy DataBase
DR HAGIS	Diabetic Retinopathy, Hypertension, Age-related macular degeneration and Glaucoma Images
DRiDB	Diabetic Retinopathy image DataBase
DRIVE	Digital Retinal Images for Vessel Extraction
Eye PACS	Eye Picture Archive Communication System
F-DCVP	Fundus-Data Computer Vision Project
HEI-MED	Hamilton Eye Institute Macular Edema Dataset
HRF	High-Resolution Fundus Segmentation
IDRID	Indian Diabetic Retinopathy Image Dataset
JSIEC	Joint Shantou International Eye Center
ROC	Online Challenge
STARE	Structured Analysis of the Retina
