# Supplementary material for: Comprehensive Review of Open-Source Fundus Image Databases for Diabetic Retinopathy Diagnosis
Source: Sensors (Basel). 2025 Sep 11;25(18):5658. doi: 10.3390/s25185658 (PMC12473202; doi:10.3390/s25185658)
Supplement: Supplementary file 1 [file sensors-25-05658-s001.zip › sensors-3769055-supplementary.pdf]

Review

Supplementary Material: Boxplots and Histograms computed for each dataset

Valérian Conquer [1,‡], Thomas Lambolais[2,*], Gustavo Andrade-Miranda[3,‡] *and* Baptiste Magnier[4,*,†]

1 valerian.conquer@etu.mines-ales.fr
2 thomas.lambolais@mines-ales.fr
3 gustavo.andrade-miranda@mines-ales.fr
4 baptiste.magnier@mines-ales.fr
* EuroMov Digital Health in Motion, Univ Montpellier, IMT Mines Ales, Ales, France.
† Service de Médecine Nucléaire, Centre Hospitalier Universitaire de Nîmes, Université de Montpellier, Nîmes, France
‡ IMT Mines Ales, Alès, France

Figures

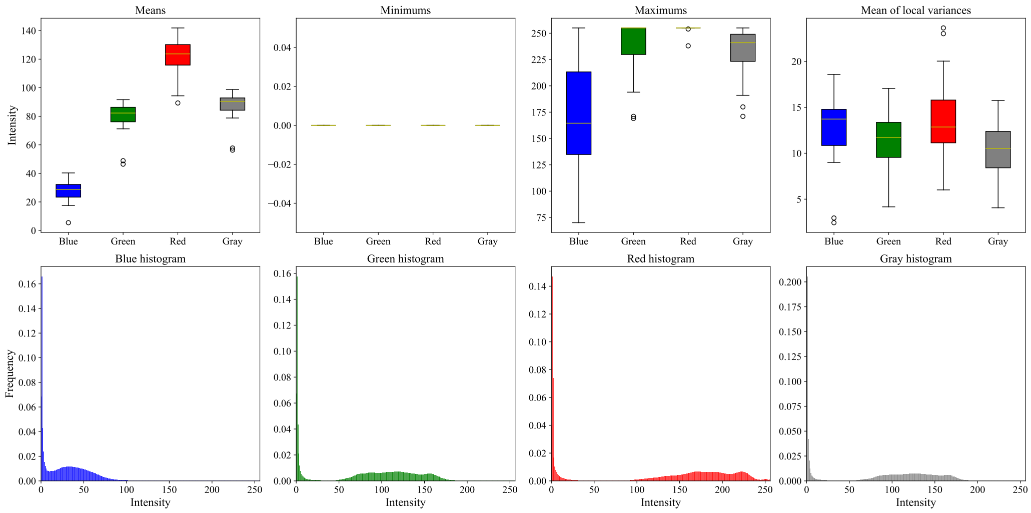

Figure S1. Average color histograms (RGB and grayscale) and box plots of key statistical features for all images in the **AGAR300** dataset, displayed separately by color channel.

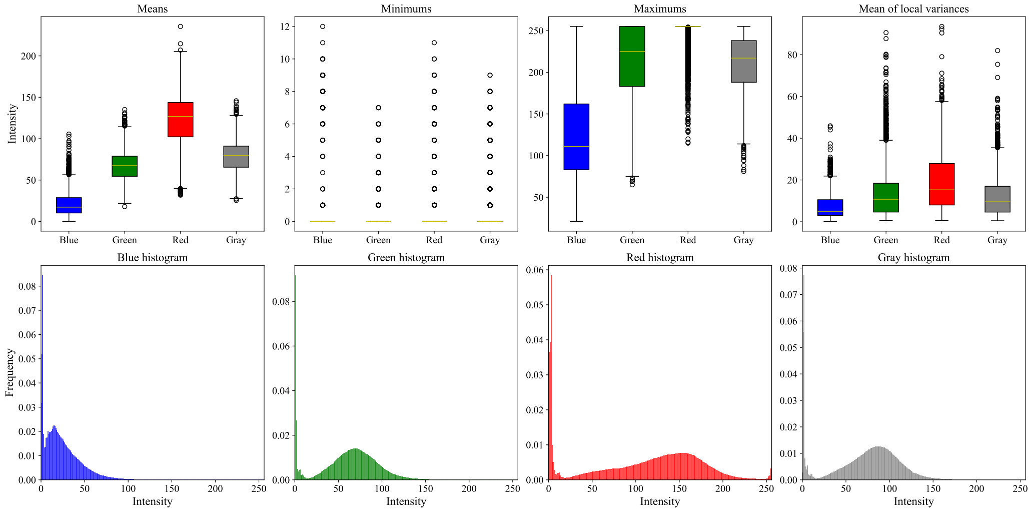

Figure S2. Average color histograms (RGB and grayscale) and box plots of key statistical features for all images in the **APTOS** dataset, displayed separately by color channel.

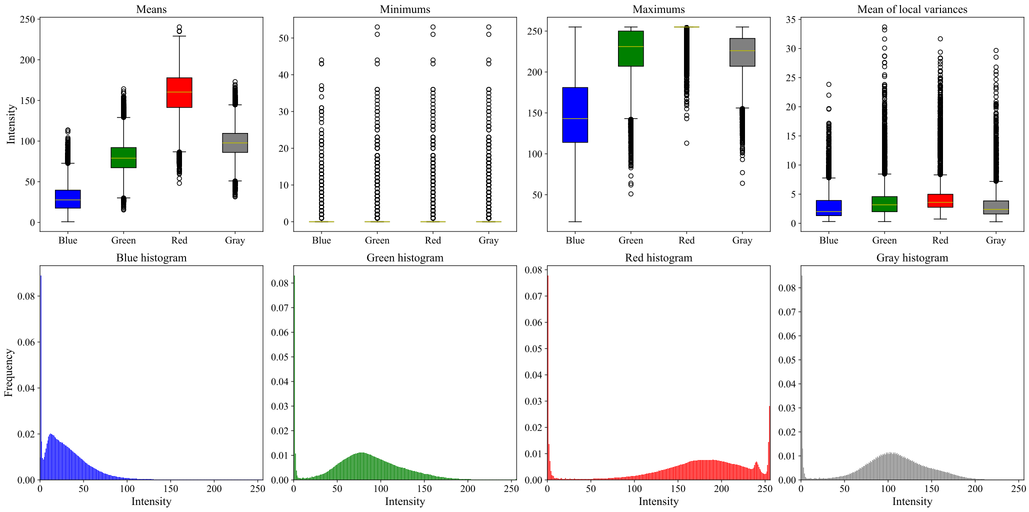

Figure S3. Average color histograms (RGB and grayscale) and box plots of key statistical features for all images in the **BRSET** dataset, displayed separately by color channel.

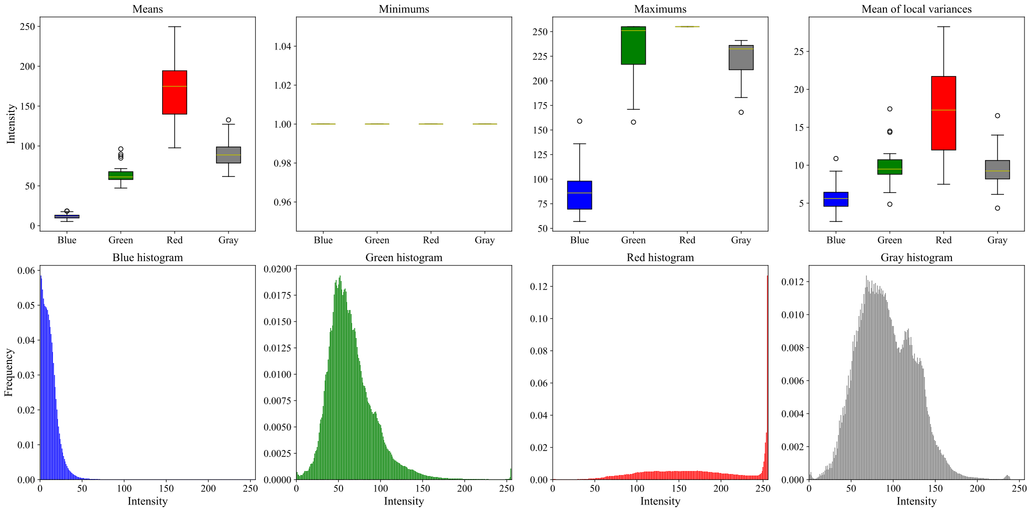

Figure S4. Average color histograms (RGB and grayscale) and box plots of key statistical features for all images in the **CHASE DB1** dataset, displayed separately by color channel.

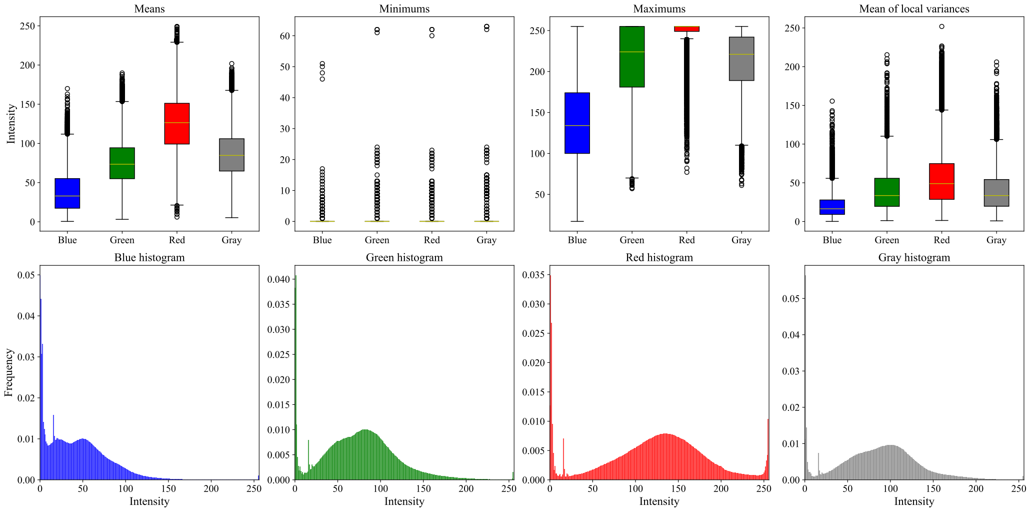

Figure S5. Average color histograms (RGB and grayscale) and box plots of key statistical features for all images in the **DDR** dataset, displayed separately by color channel.

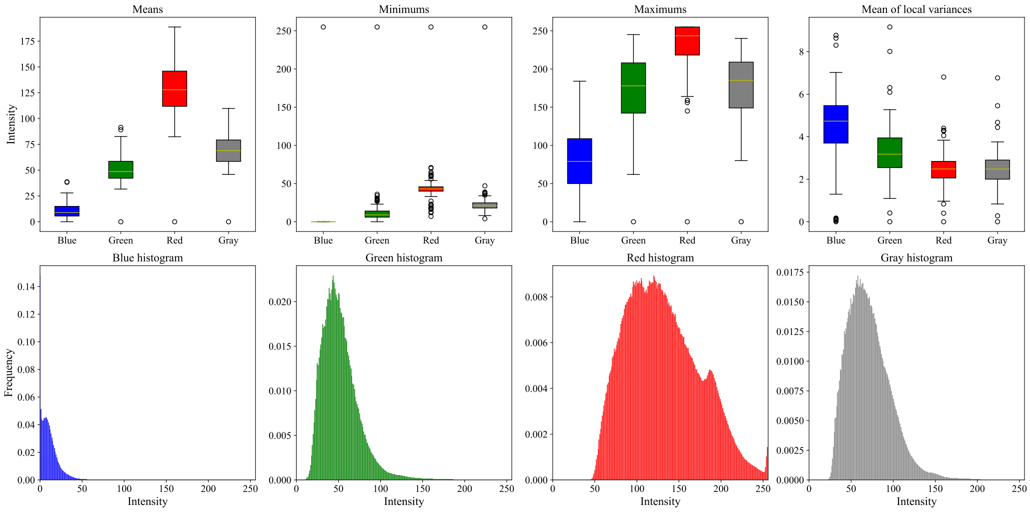

Figure S6. Average color histograms (RGB and grayscale) and box plots of key statistical features for all images in the **DiaRetDB0** dataset, displayed separately by color channel.

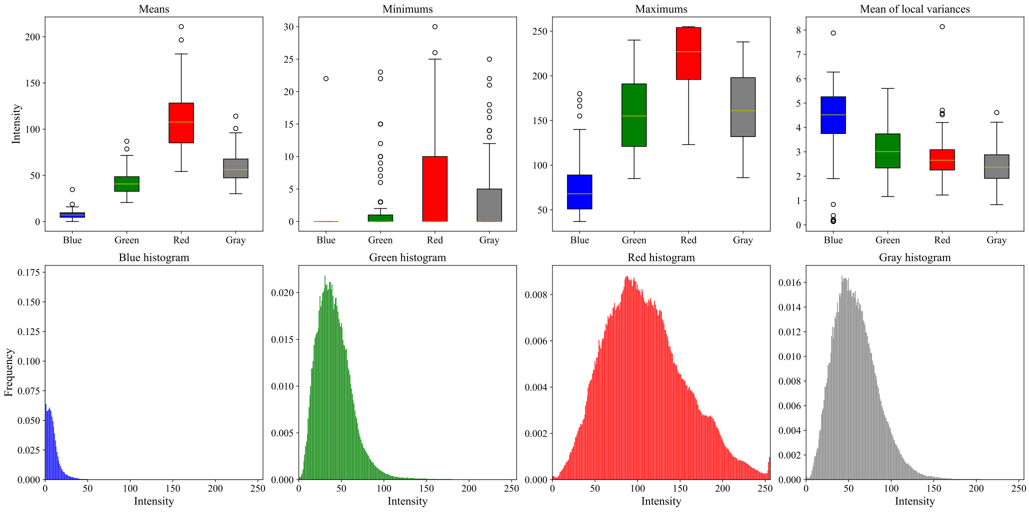

Figure S7. Average color histograms (RGB and grayscale) and box plots of key statistical features for all images in the **DiaRetDB1** dataset, displayed separately by color channel.

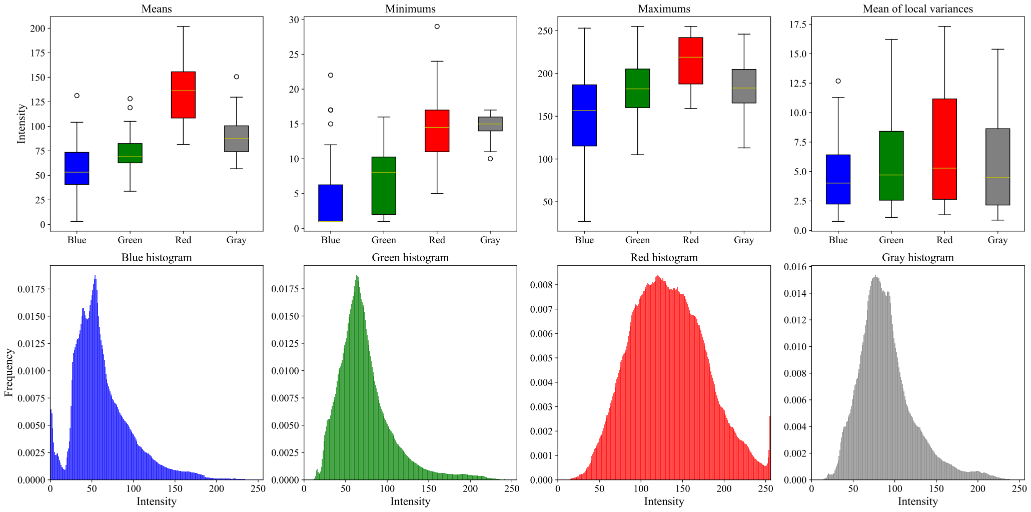

Figure S8. Average color histograms (RGB and grayscale) and box plots of key statistical features for all images in the **DR HAGIS** dataset, displayed separately by color channel.

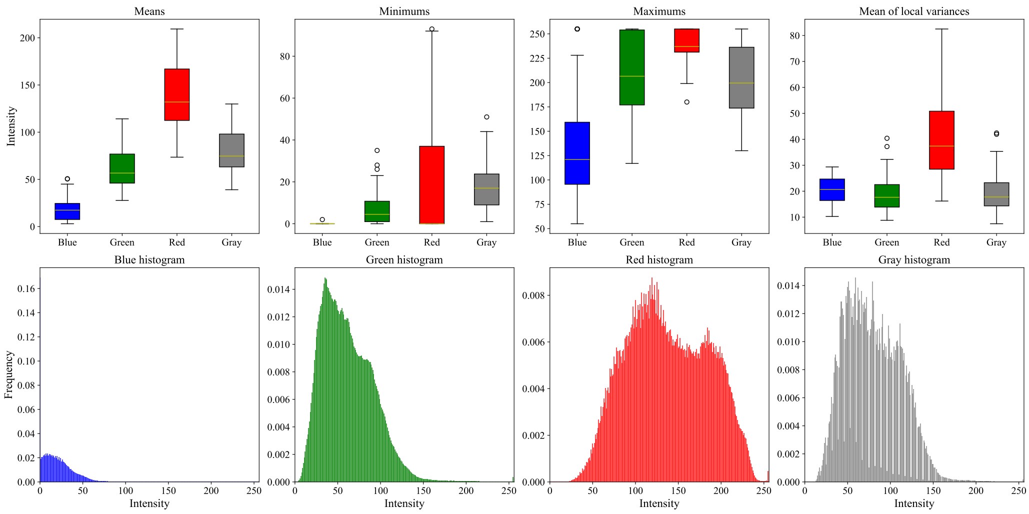

Figure S9. Average color histograms (RGB and grayscale) and box plots of key statistical features for all images in the **DRiDB** dataset, displayed separately by color channel.

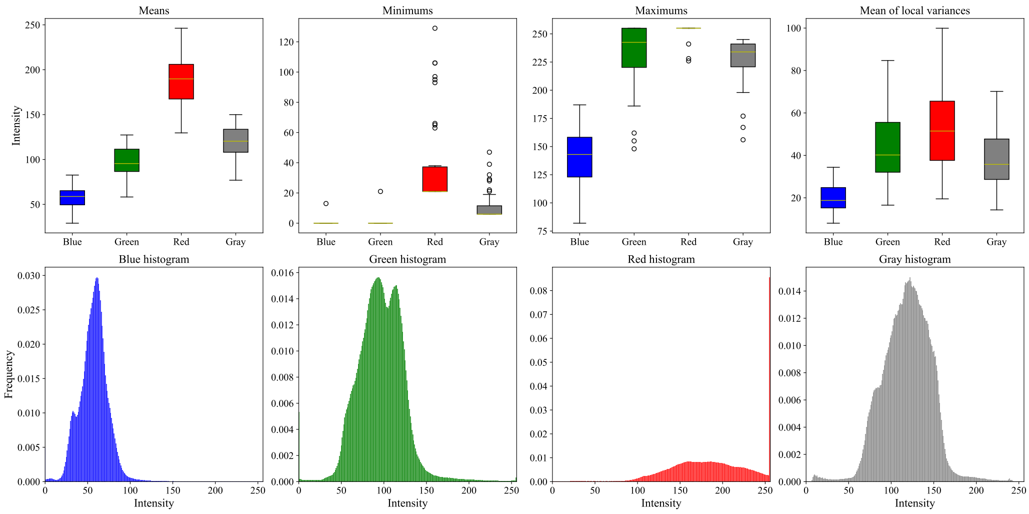

Figure S10. Average color histograms (RGB and grayscale) and box plots of key statistical features for all images in the **DRIVE** dataset, displayed separately by color channel.

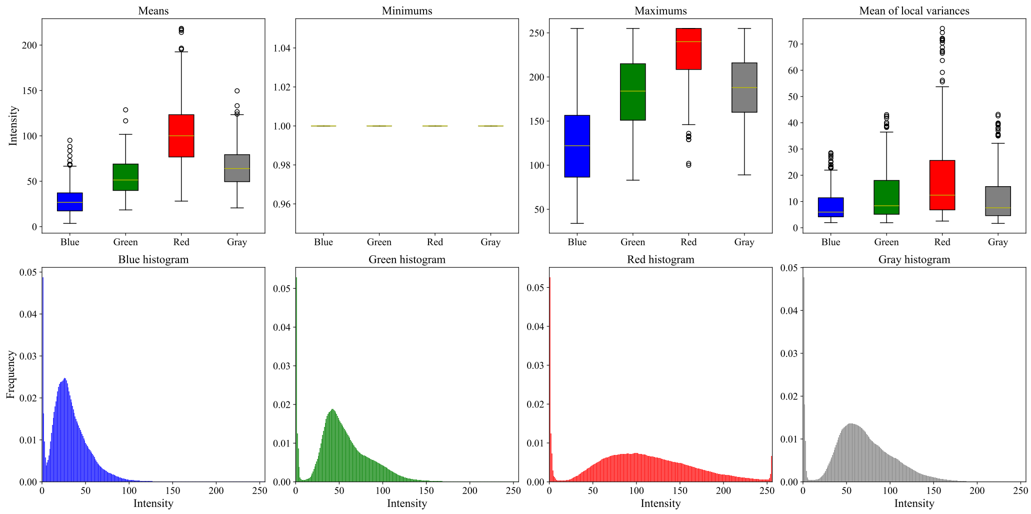

Figure S11. Average color histograms (RGB and grayscale) and box plots of key statistical features for all images in the **E-Ophtha** dataset, displayed separately by color channel.

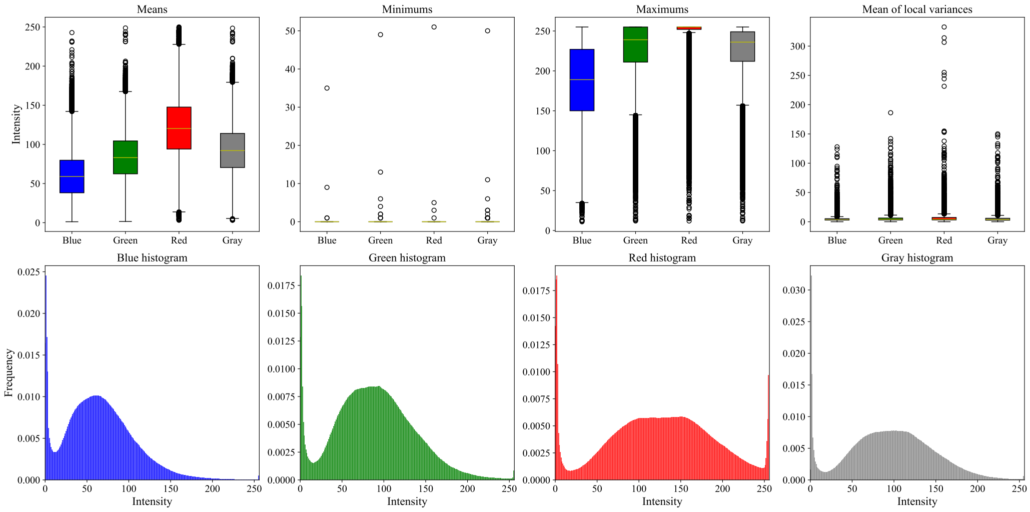

Figure S12. Average color histograms (RGB and grayscale) and box plots of key statistical features for all images in the **Eye PACS** dataset, displayed separately by color channel.

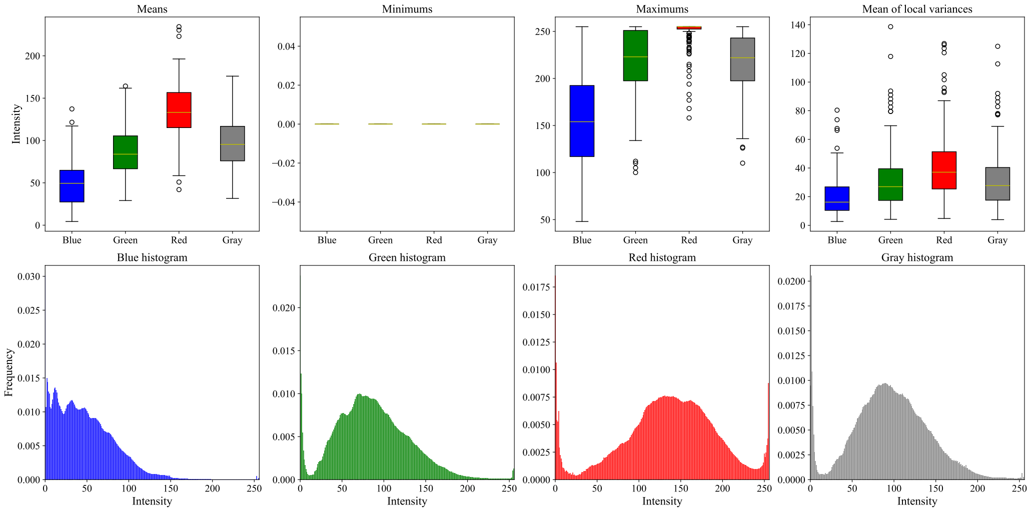

Figure S13. Average color histograms (RGB and grayscale) and box plots of key statistical features for all images in the **F-DCVP** dataset, displayed separately by color channel.

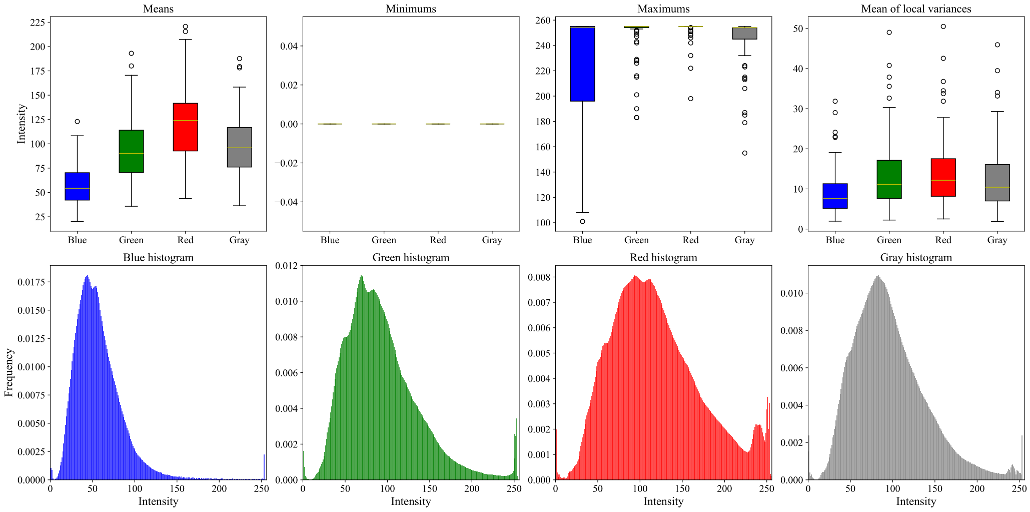

Figure S14. Average color histograms (RGB and grayscale) and box plots of key statistical features for all images in the **HEI MED** dataset, displayed separately by color channel.

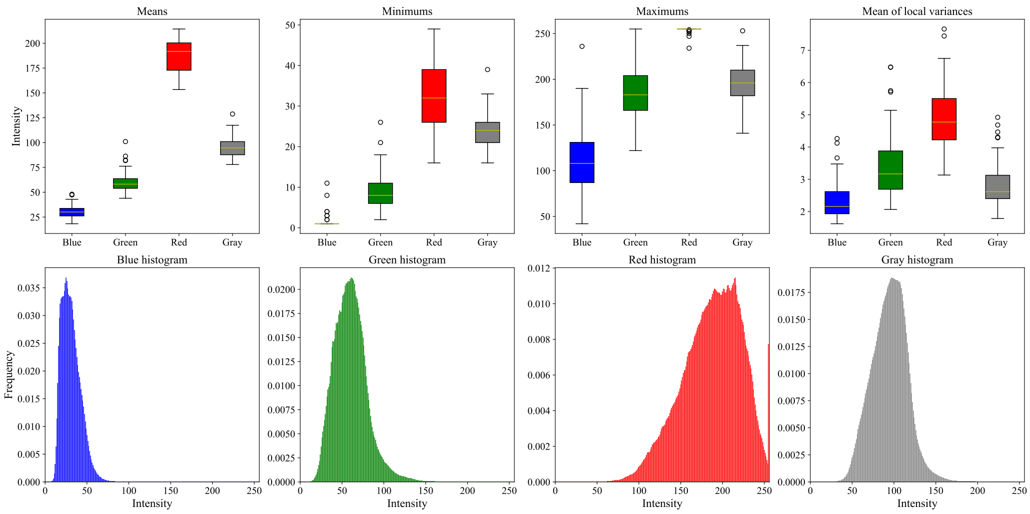

Figure S15. Average color histograms (RGB and grayscale) and box plots of key statistical features for all images in the **HRF Segmentation** dataset, displayed separately by color channel.

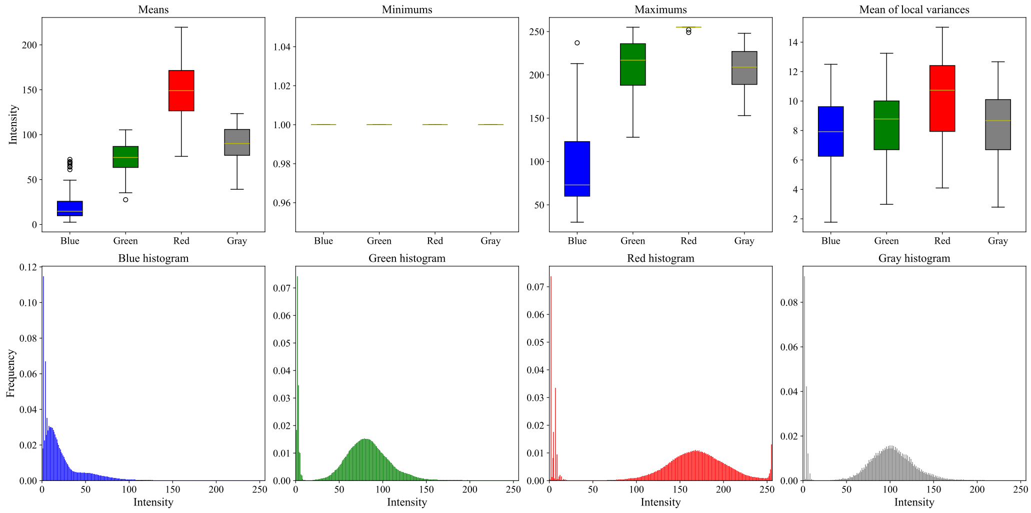

Figure S16. Average color histograms (RGB and grayscale) and box plots of key statistical features for all images in the **IDRID** dataset, displayed separately by color channel.

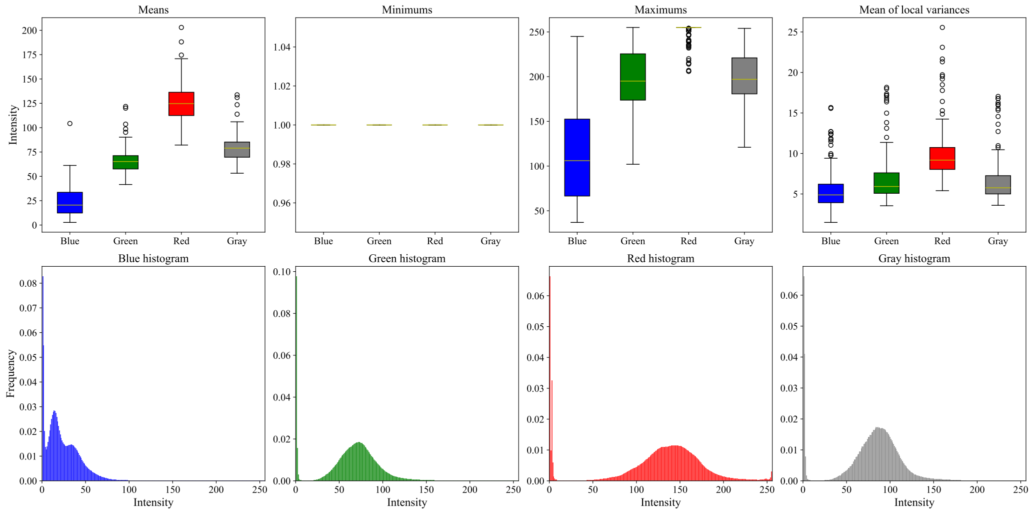

Figure S17. Average color histograms (RGB and grayscale) and box plots of key statistical features for all images in the **JSIEC** dataset, displayed separately by color channel.

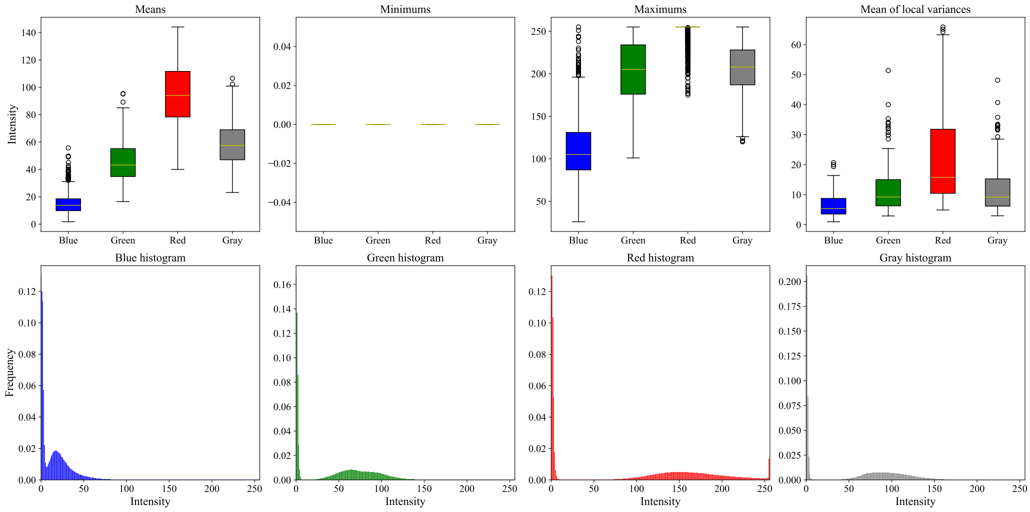

Figure S18. Average color histograms (RGB and grayscale) and box plots of key statistical features for all images in the **MESSIDOR** dataset, displayed separately by color channel.

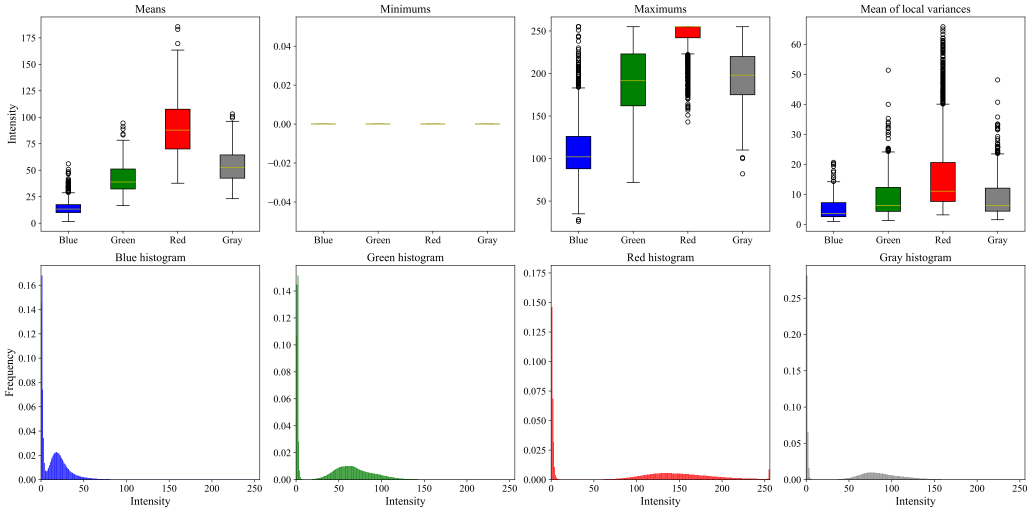

Figure S19. Average color histograms (RGB and grayscale) and box plots of key statistical features for all images in the **MESSIDOR2** dataset, displayed separately by color channel.

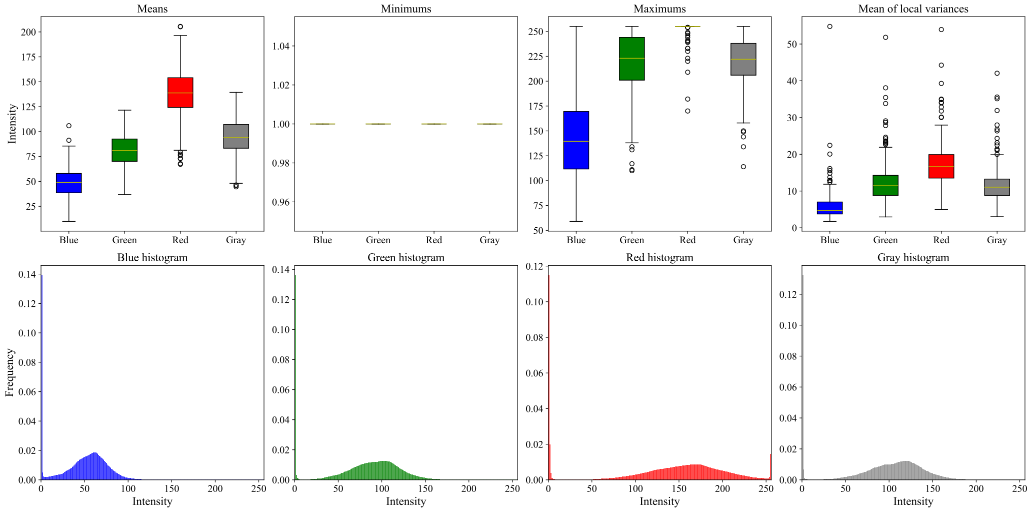

Figure S20. Average color histograms (RGB and grayscale) and box plots of key statistical features for all images in the **Retina** dataset, displayed separately by color channel.

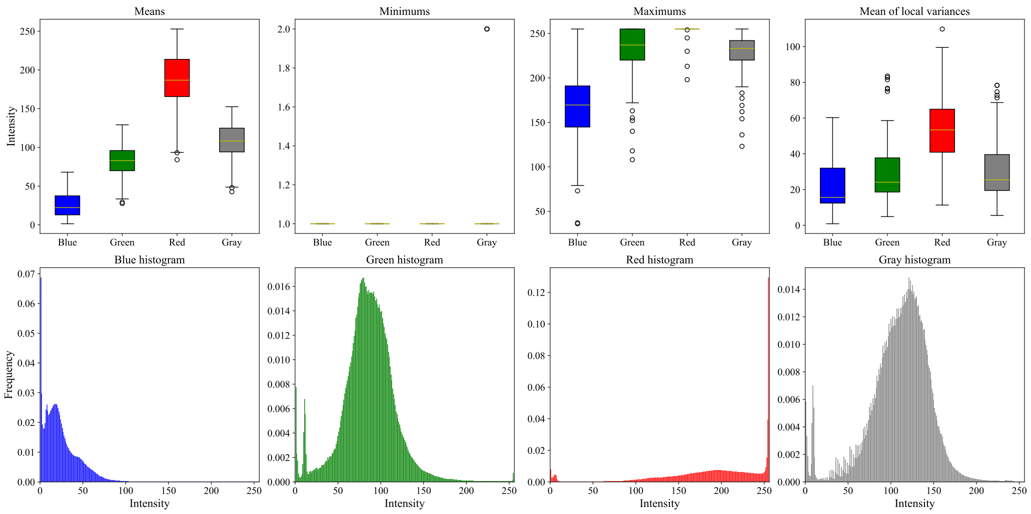

Figure S21. Average color histograms (RGB and grayscale) and box plots of key statistical features for all images in the **ROC** dataset, displayed separately by color channel.

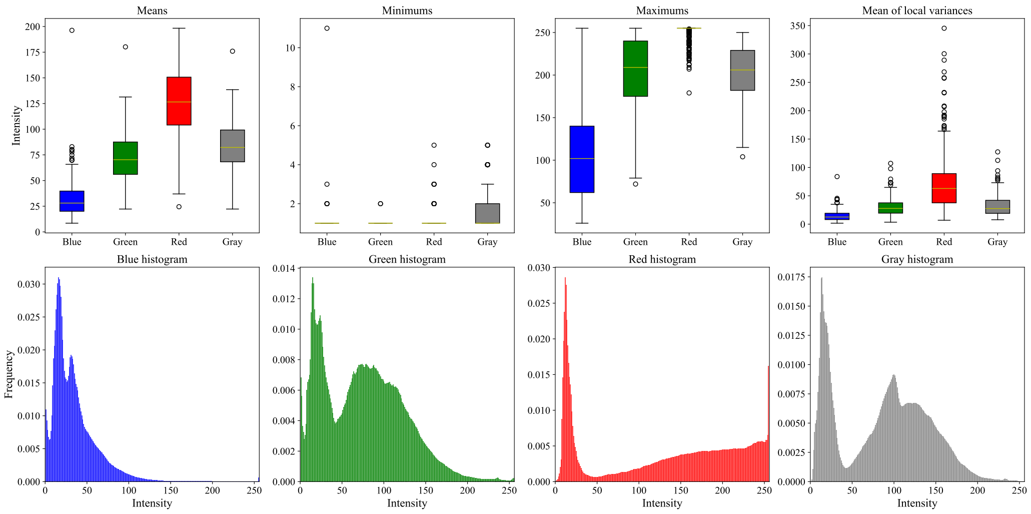

Figure S22. Average color histograms (RGB and grayscale) and box plots of key statistical features for all images in the **STARE** dataset, displayed separately by color channel.
